# Annexin A6 controls multi-organelle contact site formation and endolysosomal positioning, and remodels the STARD3 interactome

**DOI:** 10.1016/j.isci.2026.116387

**Published:** 2026-06-15

**Authors:** Marc Bernaus-Esqué, Yangjing Liu, Eva Prats, Josep M. Estanyol, Gemma Martin, Maria Calvo, Panagiota Areti Gigourtsi, Mai Khanh Linh Nguyen, Alejandra R. Álvarez, Silvana Zanlungo, Neus Agell, Albert Lu, Francesc Tebar, Carlos Enrich, Thomas Grewal, Carles Rentero

**Affiliations:** 1 Departament de Biomedicina, Unitat de Biologia Cel·lular, Facultat de Medicina i Ciències de la Salut, Universitat de Barcelona, Barcelona, Spain; 2 Centres Científics i Tecnològics (CCiT/UB) Unitats de Proteòmica, Microscòpia Òptica Avançada i Microscòpia Electrònica, Campus Casanova, Facultat de Medicina i Ciències de la Salut, Universitat de Barcelona, Barcelona, Spain; 3 School of Pharmacy, Faculty of Medicine and Health, University of Sydney, Sydney, NSW, Australia; 4 Laboratory of Cell Signalling, Department of Cellular and Molecular Biology, Biological Sciences Faculty, CARE UC, Pontificia Universidad Católica de Chile, Santiago, Chile; 5 Department of Gastroenterology, Faculty of Medicine, Pontificia Universidad Católica de Chile, Santiago, Chile; 6 Fundació de Recerca Clínic Barcelona - Institut d’Investigacions Biomèdiques August Pi i Sunyer (FRCB-IDIBAPS) Barcelona, Barcelona, Spain

**Keywords:** Molecular biology, Cell biology

## Abstract

Annexin A6 (ANXA6) regulates cholesterol transfer across membrane contact sites (MCSs) between late endosomes/lysosomes (LE/Lys) and the endoplasmic reticulum (ER) via the late endosomal StAR-related lipid transfer domain-3 (STARD3) transporter. Here, we describe a significant reduction of MCSs in ANXA6-depleted HeLa cells, which could be rescued by restoration of ANXA6 expression. Using AnxA6 as bait in BioID-based assays, we demonstrate that ANXA6 interacts with various tethers and bona fide MCS proteins that can modulate multi-organelle contacts. STARD3 interactors identified in BioID assays include the mitochondrial translocator protein (TSPO) and myosin heavy chain 9 (MYH9). Strikingly, reduced MCS formation in ANXA6-depleted cells was associated with changes in the STARD3 interactome that indicate altered MCS tethering functions of STARD3. Specifically, ANXA6 deficiency correlated with (1) altered positioning of STARD3-positive LE/Lys; (2) a new repertoire of cortical actin-binding proteins, including myosins interacting with STARD3; (3) and decreased microvillar structures and focal adhesions.

## Highlights

• ANXA6 depletion reduces membrane contact sites in HeLa and RPE cells
• Novel interactors of StARD3 include mitochondrial TSPO and myosin MHY9
• ANXA6 depletion alters StARD3 tethering and positioning of StARD3-positive LE/Lys

## Graphical abstract

**Figure:**
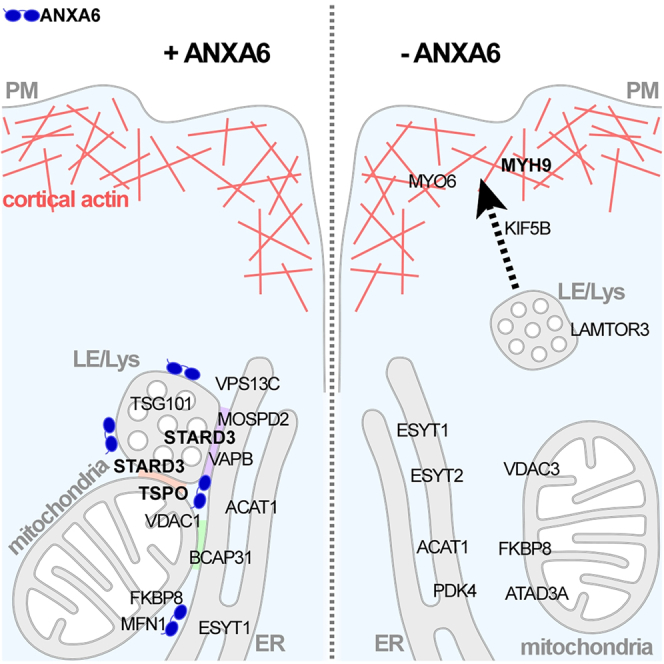

## Introduction

Cellular compartments are no longer regarded as independent entities but rather are characterized by inter-organelle communication to integrate information and perform coordinated cellular functions.^1^ This is established via vesicular transport and membrane trafficking or through the formation of membrane contact sites (MCSs) between compartments.^2^ Significantly, the latter concept incorporates organelles that were initially excluded from vesicular trafficking pathways, such as mitochondria and peroxisomes.

Recent research yielded a catalog of MCS-associated proteins, bridging the endoplasmic reticulum (ER), endosomes, lysosomes, mitochondria, lipid droplets, Golgi, peroxisomes, plasma membrane (PM), or the nucleus for intra- and inter-organelle communication.^3^ Some MCS proteins act as tethers, while others perform transport functions mostly related to lipid or ion transfer across MCSs.^1^^,^^4^^,^^5^ However, it remains unknown if universal/common players exist that could coordinate the overall formation and functioning of MCSs.

Annexins are a family of proteins that bind to membranes in a Ca^2+^-dependent manner,^6^^,^^7^ and three annexins have been identified in MCSs, ANXA1, ANXA6, and ANXA11.^8^^,^^9^^,^^10^^,^^11^ They can all bind one or two membranes simultaneously to possibly act as tethers or scaffolds that could bring membranes of neighboring organelles in close proximity. In fact, annexins also bind cholesterol, which likely adds to their ability to operate at the interface between organelles.^12^ Furthermore, all human annexins harbor a variant of the two phenylalanines (FF) in an acidic tract (FFAT) motif, FFNT (two phenylalanines in a neutral tract),^13^^,^^14^^,^^15^ both of which are well-established motifs with crucial roles in MCS formation. Therefore, considering their abundance, multiple cellular locations, promiscuity, and innate properties, a function for certain annexins in MCS formation appears reasonable.^3^^,^^7^^,^^8^^,^^15^^,^^16^^,^^17^

Over the years, we and others have shown that ANXA6 participates in various cellular activities, such as cholesterol uptake and export from late endosomes/lysosomes (LE/Lys), trafficking of Ca^2+^ channels to the PM, modulation of the cytoskeleton, PM repair, and signal complex assembly/disassembly through direct interactions with regulatory or signaling proteins and receptors.^7^^,^^8^^,^^17^

Here, we investigated the role of ANXA6 in MCS formation between LE/Lys, ER, and mitochondria by transmission electron microscopy (TEM) and proximity ligation assays (PLAs). Strikingly, TEM and PLA revealed that depletion of ANXA6 affected the length and the number of MCSs between the LE/Lys and ER, LE/Lys and mitochondria, and ER and mitochondria.

To further study the effects of ANXA6 on MCS formation, the LE cholesterol transporter StAR-related lipid transfer domain-3 (STARD3), a well-known MCS tether between LE/Lys and the ER and possibly between the LE/Lys and mitochondria,^18^^,^^19^^,^^20^^,^^21^ was selected for further analysis. Given that we previously demonstrated ANXA6 depletion to influence MCS formation in cells lacking the Niemann-Pick type C1 (NPC1) cholesterol transporter and enable STARD3-mediated cholesterol transfer between LE/Lys and the ER,^22^ we hypothesized that ANXA6 deficiency could influence STARD3 location and interacting protein networks. We, therefore, compared the STARD3 interactomes in control and ANXA6-depleted HeLa cells. Notably, we identified the interaction of STARD3 with the mitochondrial translocator protein (TSPO), which was less pronounced in ANXA6-depleted HeLa cells, indicating ANXA6 to regulate the STARD3/TSPO tethering complex at LE/Lys-mitochondria contact sites. In ANXA6-deficient cells, reduced MCS numbers coincided with a loss of STARD3 interaction with ER-associated proteins such as vesicle-associated membrane protein-associated protein B (VAPB) and motile sperm domain containing 2 (MOSPD2). These changes in the STARD3 interactome upon ANXA6 depletion indicated alterations in the MCS tethering function of STARD3 and correlated with (1) alterations in the positioning of STARD3-positive LE/Lys; (2) an increased number and new repertoire of cortical actin-binding proteins, including myosins, interacting with STARD3; and (3) structural changes affecting microvilli and focal adhesion (FA) numbers, indicating rearrangement of PM organization.

Taken together, here we demonstrate that the presence (formation) of MCSs between LE/Lys, the ER, and mitochondria is regulated in an ANXA6-dependent manner, influencing the localization and site-specific protein interactions of STARD3, which are crucial for maintaining cellular homeostasis.

## Results

### ANXA6 depletion reduces membrane contact site formation in HeLa cells

**Figure 1:**
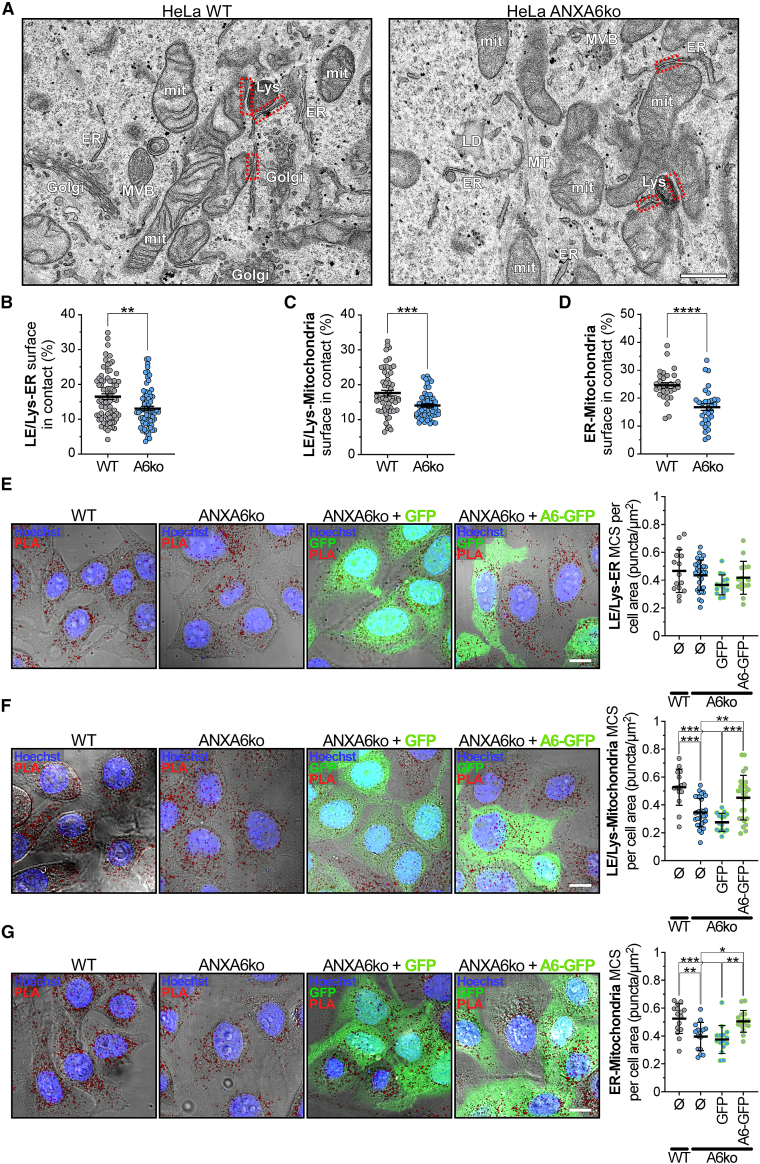
Loss of ANXA6 reduces LE/Lys contacts with ER and mitochondria (A) Representative TEM images of HeLa WT and ANXA6ko cells showing the ultrastructural organization of the endoplasmic reticulum (ER), lysosomes (Lys), mitochondria (mit), multivesicular bodies (MVB), lipid droplets (LD), and Golgi apparatus. Red dashed squares highlight MCSs between organelles. Scale bar, 400 nm. (B–D) Quantification of LE/Lys-ER (B), LE/Lys-mitochondria (C), and ER-mitochondria (D) contact site length in WT and ANXA6ko HeLa cells (*n* ≥ 30 cells). (E–G) PLA analysis and quantification of the number of MCSs between LE/Lys-ER (E), LE/Lys-mitochondria (F), and ER-mitochondria (G) in WT, ANXA6ko, ANXA6ko transfected with GFP, and ANXA6ko transfected with ANXA6-GFP HeLa cells (*n* > 20 cells, from 2 independent measurements). **∅**, Untransfected cells. Scale bars, 10 μm. Data are represented as mean ± SEM. ∗*p* < 0.05, ∗∗*p* < 0.01, ∗∗∗*p* < 0.001, ∗∗∗∗*p* < 0.0001.

We previously demonstrated ANXA6 depletion to alleviate cholesterol accumulation in LE/Lys of NPC1-mutant CHO cells. This was associated with increased LE/Lys-ER membrane contacts and STARD3-mediated cholesterol transfer to the ER for esterification and subsequent storage in lipid droplets,^22^ suggesting ANXA6 as LE/Lys-ER contacts regulator. Here, we investigated a potential wider role for ANXA6 in MCS formation in HeLa cells with functional NPC1 and compared membrane contacts of three pairs of organelles, LE/Lys-ER, LE/Lys-mitochondria, and ER-mitochondria, in wild-type (HeLa-WT) cells and using CRISPR-Cas9 technology, in ANXA6-depleted cells (HeLa-ANXA6ko) (Figure 1; Figure S1A).

A flat embedding technique was used for TEM analysis,^23^ and grids with sections (60–70 nm) from three independent experiments were used to acquire images to examine and quantify MCSs (≈5–40 nm). Figure 1A shows the representative images of WT and ANXA6ko cells with comparable ultrastructure, morphology, size and organization of major cellular compartments. Both cell lines displayed a morphological diversity of LE (including multivesicular bodies/lysosomal structures).^24^^,^^25^^,^^26^^,^^27^ Additionally, non-significant perimeter changes in LE/Lys and mitochondria were observed (Figures S1B and S1C).

Quantitative analysis demonstrated a significant decrease of the extent of MCSs (percentage of surface in contact) between LE/Lys and ER, LE/Lys and mitochondria, as well as ER and mitochondria in ANXA6ko compared to WT cells (Figures 1B–1D). Importantly, these alterations of inter-organelle contacts in ANXA6-deficient HeLa cells were also evident in PLAs, showing reduced number of contacts per cell area between LE/Lys-mitochondria and ER-mitochondria (Figures 1E–1G, control of antibodies in Figure S2). While the quantification of PLAs confirmed the TEM analysis in regard to reduced contacts between LE/Lys-mitochondria and ER-mitochondria upon ANXA6 depletion, PLAs showed similar numbers of LE/Lys-ER contacts in ANXA6-depleted and control cells and TEM analysis showed a reduced length of MCS in ANXA6-depleted HeLa cells (compare Figures 1B and 1E).

To determine if restoration of ANXA6 expression could elevate the reduced MCS numbers in cells lacking ANXA6, HeLa-ANXA6ko cells were transiently transfected with fluorescently tagged ANXA6 (ANXA6-GFP) or GFP (control) alone. After 24 h, cells were fixed and incubated with specific antibodies followed by PLA reagents. PLA puncta were quantified using an ImageJ2 macro that normalized the puncta per cellular area (see STAR Methods for details). Strikingly, the transient restoration of ANXA6 protein expression in ANXA6ko cells revealed a significant recovery of MCS numbers comparable to WT controls between LE/Lys-mitochondria (Figure 1F) and mitochondria-ER (Figure 1G). On the other hand, transient restoration of ANXA6 expression in ANXA6ko cells did not impact the number of PLA puncta between LE/Lys and the ER (Figure 1E). Hence, based on TEM data, the presence or absence of ANXA6 fundamentally regulates the extent and number of MCSs that are formed between the two major organelles associated with the ER: mitochondria and LE/Lys.

**Figure 2:**
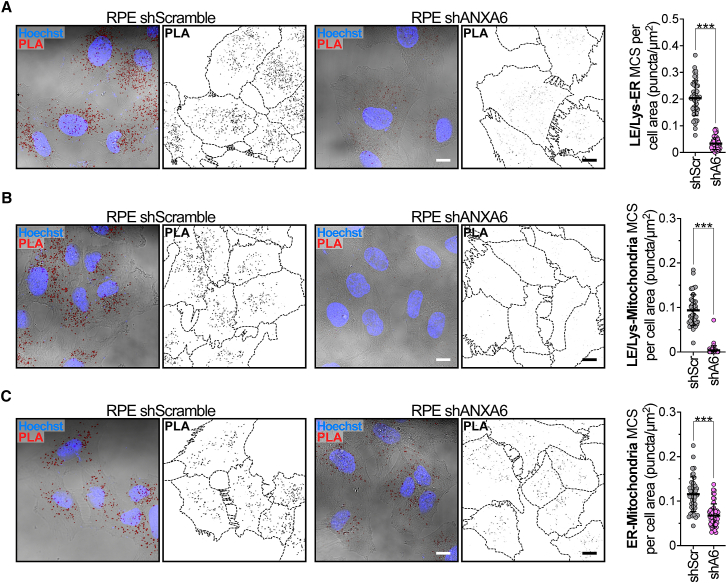
Depletion of ANXA6 in RPE cells decreases the number of membrane contact sites PLA analysis and quantification of the number of MCSs between LE/Lys-ER (A), LE/Lys-mitochondria (B), and ER-mitochondria (C) in shRNA Scramble and shANXA6-infected RPE cells (*n* > 50 cells, from 2 independent measurements). Scale bars, 10 μm. Data are represented as mean ± SEM. Statistical significance was calculated using a two-tailed Mann-Whitney test. ∗∗∗*p* < 0.001.

To ascertain that ANXA6 deficiency also decreased the MCS in other cell lines, non-tumor human retinal pigment epithelium (RPE) cells^28^ expressing Scramble short hairpin RNA (shRNA) (RPE-shScramble) and a stable ANXA6-knockdown RPE cell line (RPE-shANXA6) were compared (Figure S1A). Indeed, PLA quantification showed that ANXA6 knockdown in RPE cells significantly decreased the three MCS subsets studied (Figure 2). Hence, ANXA6 is part of a general molecular machinery regulating MCS number and size between various organelles, and its depletion alters the wiring of organelle contacts.

### ANXA6 influences crosstalk of STARD3 with proteins located in membrane contact sites, the cortical actin-cytoskeleton, and intracellular trafficking pathways

The above findings indicated ANXA6 depletion to interfere with the interaction of tethers that couple organelles**.** To provide a more comprehensive picture of ANXA6 protein-protein interactions and offer innovative insights into MCS formation and dynamics, we next determined the ANXA6 interactome.

**Figure 3:**
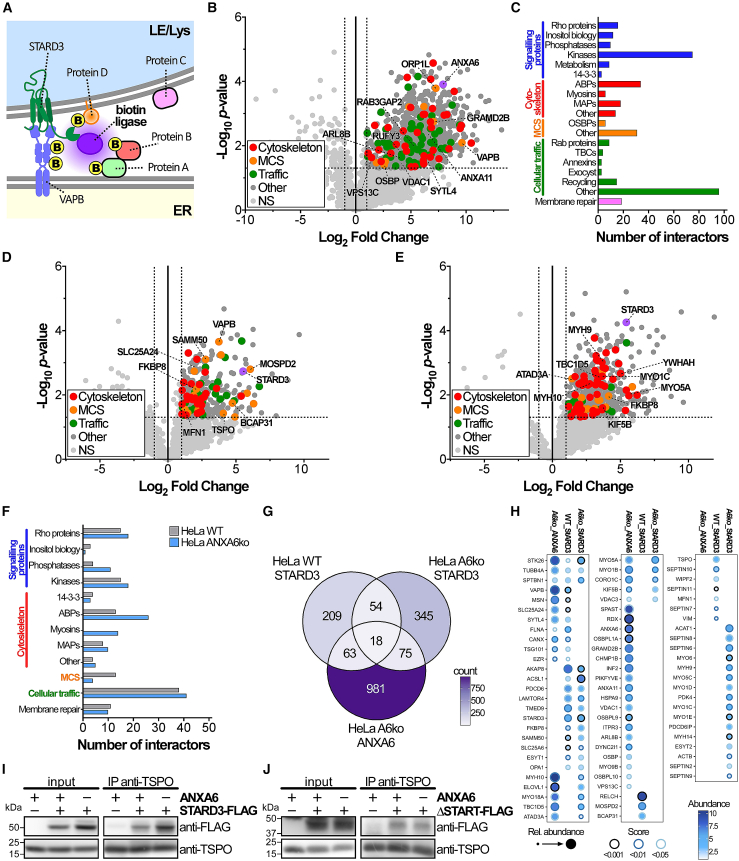
ANXA6 modulates the STARD3 interactome (A) Schematic representation of the proximity-dependent biotinylation (PDB) assay of STARD3. A similar strategy was used with ANXA6 as bait. (B and C) Volcano plot (B) and functional categorization (C) of the ANXA6 PDB interactors produced in ANXA6ko HeLa cells colored by biological process (cytoskeleton [red], MCS [orange], cellular trafficking [green], and others [dark gray]). (D–F) Volcano plots (D and E) and functional categorization comparison (F) of STARD3 PDB interactomes in WT (D) vs. ANXA6ko (E) HeLa cells. (G) Venn diagram showing the number of overlapping identified proteins of ANXA6 and STARD3 PDB interactomes in WT and ANXA6ko HeLa cells. (H) ProHit-viz of selected ANXA6 and STARD3 proximity interactors in WT and ANXA6ko HeLa cells (see Tables S1–S4 for complete dataset). (I and J) Representative co-immunoprecipitation assays of TSPO with STARD3-FLAG (G) or STARD3-ΔSTART-FLAG (H) in ANXA6-expressing or -deficient HeLa cells (*n* = 2 independent measurements).

Biotin-ligase UltraID fused to ANXA6 (Figures 3A and S3) served as bait,^29^ and to avoid competition with endogenous ANXA6, studies were performed in ANXA6ko cells. Lysates from transfected and biotin-treated cells were analyzed by a liquid chromatography-mass spectrometry-coupled PDL screen. To consider a hit as an ANXA6 interactor, the statistical analysis was based on a *p* value = 0.05 (−log_10_(p) ≈ 1.30103) and fold-change (FC) = 2 (log_2_(FC) = ± 1), identifying 1,140 hits including previously described RAF1, TBC1D5, or SPTBN1.^30^^,^^31^^,^^32^

String Database Protein Ontology revealed several major groups of ANXA6 interactors that according to their function were classified into signaling, cytoskeletal, MCS-associated proteins, and those involved in membrane trafficking pathways (Figure 3C). In line with the multiple ANXA6 localizations,^8^ the most relevant ANXA6 interactors were cytoplasmic or associated with the cortical cytoskeleton, mitochondria, LE/Lys, and PM.

We next focused on two groups of ANXA6 interactors, and the top hits of (1) well-defined *bona fide* proteins located in MCS and (2) actomyosin- and tubulin-associated proteins are shown in Figures 3B and 3C. Other ANXA6 interactors that were not analyzed further included proteins associated with trafficking pathways, signaling proteins, and proteins of the membrane repair kit.

Within the *bona fide* proteins located in MCSs, we identified several ANXA6 interactors, some of those acting as tethers (VAPB and ANXA11^3^) or being involved in the transport of cholesterol, lipid (ORP1L, OSBP, and GRAMD2B), or Ca^2+^ (BCAP31, VDAC1, and ITPR1) across MCSs (Figure 3B). Previously we already validated ANXA6 interacting with TBC1D15.^22^

In addition, the proteomic screen identified ANXA6 interactors essential for intracellular trafficking, as well as numerous actin-binding proteins including myosins, all of which were implicated in the formation and/or dynamics of LE/Lys-ER and LE/Lys-mitochondria contacts.

Given ANXA6 depletion to reduce MCS formation (Figures 1 and 2), ANXA6 interacting with MCS-associated tethers and lipid transfer proteins (Figure 3B, 3C, and 3H; Table S2), and ANXA6 modulating the cholesterol transfer ability of STARD3 across LE/Lys-ER contacts,^22^ we hypothesized that ANXA6 could influence overall STARD3 location, activity, and interacting protein networks, possibly within MCSs.

Therefore, we next examined the STARD3 interactomes in WT and ANXA6ko cells (Figures 3D and 3E). The miniTurboID biotin ligase^33^ was fused to STARD3 or as control, to lysosomal transmembrane protein 192 (TMEM192). In WT cells, the most prominent hits (344 proteins) using the String Database Protein Ontology analysis (Figure 3F) belonged to groups related to signaling, cytoskeleton, membrane traffic, MCSs, and membrane repair, with most hits corresponding to cytosolic proteins, followed by the endomembrane system. Several STARD3 interactors were related to membrane contacts, such as ER proteins VAPB, MOSPD2, and BCAP31, and outer mitochondrial membrane (OMM) proteins, for instance, FKBP8 or MFN1. Other mitochondrial STARD3 interactors included SAMM50, TSPO, and several large solute carrier family 25 (SLC25) mitochondrial transporters.

Interestingly, TSPO, a transmembrane protein at the OMM, was highly enriched in the STARD3 interactome of HeLa WT cells. TSPO is now known to facilitate, possibly together with STARD1 and TOMM40, cholesterol import to mitochondria.^34^^,^^35^

Remarkably, in ANXA6-depleted HeLa cells, of the 345 protein STARD3 interactors, the main ER-resident STARD3 interactors, such as VAPA/B or MOSPD2, and other ER proteins, including BCAP31 or MCS-associated proteins like ATAD3A and MFN1, were lacking (Table S2). Similarly, the interaction of STARD3 with TSPO, measured as FC ratio between TSPO abundance in STARD3 vs. TMEM192 proteomics, was much less pronounced in ANXA6ko (FC = 3.03, *p* value = 0.08) compared to WT HeLa cells (FC = 16.48, *p* value = 0.038).

STARD3 was previously described to transfer cholesterol to mitochondria in NPC disease, yet mitochondrial partner proteins possibly supporting this process have remained unknown.^21^^,^^36^^,^^37^^,^^38^^,^^39^^,^^40^ TSPO co-immunoprecipitation with ectopically expressed FLAG-tagged STARD3 (Figure 3I) or the truncated STARD3-ΔSTART-FLAG protein (Figure 3J) in WT and ANXA6ko HeLa cells further validates the described interaction and potential partnership between the two cholesterol transporter/binding proteins TSPO and STARD3.

Altogether, the reduced enrichment of MCS-associated proteins in the STARD3 interactome of ANXA6-depleted HeLa cells (Figure 3F) correlated with reduction of MCSs (LE/Lys-mitochondria and ER-mitochondria) in these cells (Figure 1), indicating that ANXA6 is a key player coordinating the formation of STARD3-containing MCSs.

As illustrated in Figure 3G, the Venn diagram shows the number of proteins for each interactome using ANXA6-ultraID and STARD3-miniTurboID. The figure is accompanied by a table that enumerates the 18 common proteins of the three interactomes analyzed in this study (Table S1). Interestingly, proteins identified in both the ANXA6 and STARD3 interactomes include several cytoskeletal proteins (TUBB4A and SPTBN1), regulators of vesicular trafficking (RAB27B and SCYL1), and modulators of endocytosis and membrane dynamics (GSK3A) (Figure 3H). These shared interactions could suggest a common functional axis that coordinates cytoskeletal organization and vesicular and membrane transport, potentially regulating lipid exchange and the dynamics of MCSs within the cell.

In addition, the loss of ANXA6 triggered a significant alteration in the overall interaction of STARD3 with cytoskeletal-related proteins (Tables S3–S5), in particular the increased interaction of STARD3 with non-muscular and unconventional myosins (e.g., MYH9, MYH10, and MYO1C) including MYO6, which mediates endosome association with cortical actin filaments^41^^,^^42^ and which was lacking in WT cells. This shift to increasingly interact with the cortical actomyosin cytoskeleton upon ANXA6 depletion was also reflected by STARD3 interactors such as coronins, tropomyosins, septins, and kinesins (including kinesin family member 5B [KIF5B]).

### ANXA6 depletion alters positioning of STARD3-containing endolysosomes

**Figure 4:**
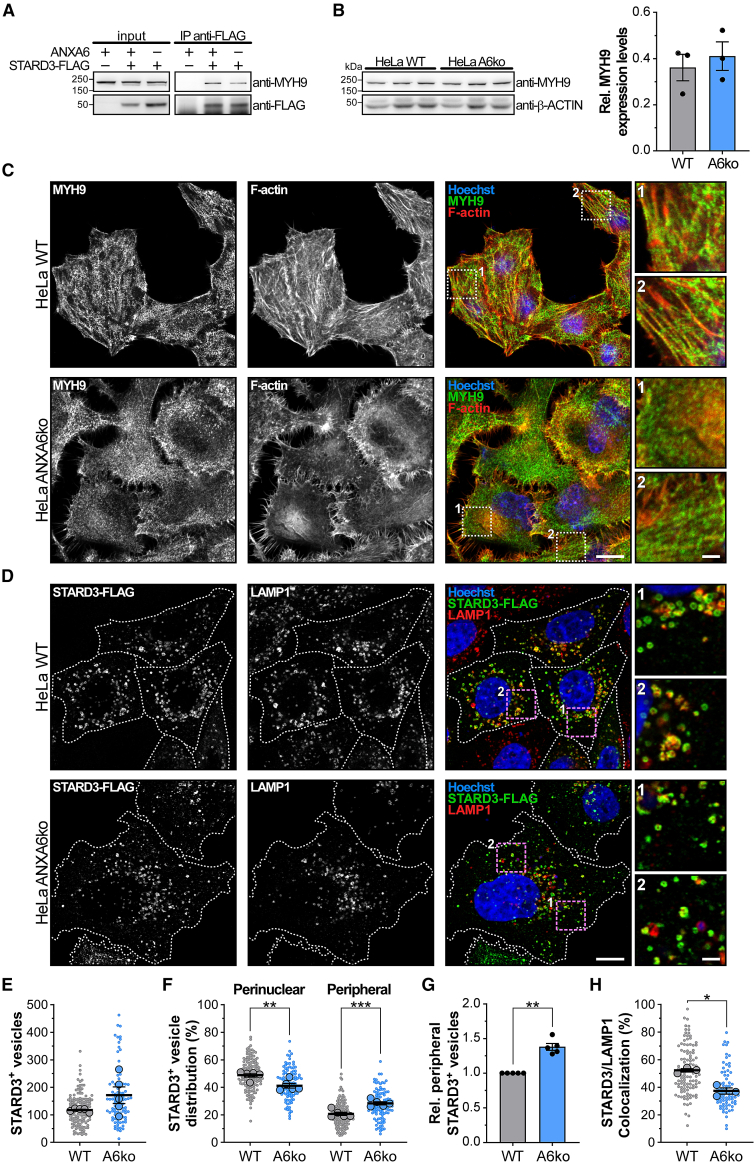
ANXA6 controls STARD3-cytoskeleton interaction and LE/Lys positioning in HeLa cells (A) Representative co-immunoprecipitation assay of MYH9 with STARD3-FLAG in ANXA6-expressing or -deficient HeLa cells (*n* = 2 independent measurements). (B) Western blot analysis of MYH9 protein expression and normalized quantification in WT and ANXA6ko HeLa cells (*n* = 3 independent measurements). (C) Confocal immunofluorescence images of MYH9 (green) and F-actin (red) in WT and ANXA6ko HeLa cells. Enlarged insets show MYH9 and actin filament staining details. (D–H) (D) Representative confocal immunofluorescence images and (E–H) quantifications of transfected STARD3-FLAG (green) vesicle number, distribution, and colocalization with LAMP1 (red) in WT and ANXA6ko HeLa cells (*n* > 50 cells, 3–5 independent measurements). Scale bars,10 μm; inset scale bars, 2 μm. Data are represented as mean ± SEM. Statistical significance was calculated using unpaired (B) and paired (E, F, and H) Student’s *t* test and two-tailed Mann Whitney test (G). ∗*p* < 0.05, ∗∗*p* < 0.01, ∗∗∗*p* < 0.001.

We next validated STARD3 interaction with MYH9 (myosin IIa), which is well-known to function in cortical actin cytoskeleton organization and FA and endosome dynamics.^43^ Indeed, MYH9 and ectopically expressed FLAG-tagged STARD3 co-immunoprecipitated in whole-cell lysates from HeLa cells (Figure 4A). These changes could not be attributed to decreased MYH9 protein levels in ANXA6ko cells (Figure 4B). Furthermore, in HeLa-WT cells, MYH9 was distributed on top of the actin stress fibers and on the cortical cytoskeleton. However, in HeLa-ANXA6ko cells, MYH9 lacked fiber distribution within the cortical region, and less actin stress fibers as well as an altered cortical actin network in ANXA6ko cells was observed (Figure 4C).

In addition to tethers, LE/Lys positioning relies on links with microtubules, actin filaments, actin-binding proteins (ABPs), and respective motor protein complexes. Indeed, besides MYH9, several hits uncovered in the ANXA6-based proximity-dependent biotinylation (PDB) assays were associated with LE/Lys positioning, such as ARL8B, RUFY3,^44^ and SYTL4, RAB3GAP2^43^; other vesicle trafficking-related myosins, such as MYO1C or MYO5A,^45^^,^^46^^,^^47^ were also present. As ANXA6 depletion reduced MCSs and triggered substantial changes in the STARD3 interactome, we speculated this could alter LE/Lys positioning. We, therefore, compared the number and cellular distribution (perinuclear vs. peripheral) of LAMP1-, CD63- (Figures 4D and S4A–S4E), and STARD3-positive LE/Lys in WT and ANXA6ko HeLa cells (Figures 4D–4H).

While LAMP1- and CD63-positive LE/Lys did not reveal changes or alterations in number, cellular distribution, or colocalization (Figures S4A–S4D), STARD3-positive LE/Lys structures differed significantly between HeLa WT and ANXA6ko cells. In fact, STARD3-positive LE/Lys in ANXA6ko cells showed an increased positioning toward the cell periphery in 38% of vesicles. This indicated that ANXA6 depletion impacted not only MCS formation but also the positioning of a subset of LE/Lys that were positive for STARD3 and located close to the PM (Figures 4F–4H).

**Figure 5:**
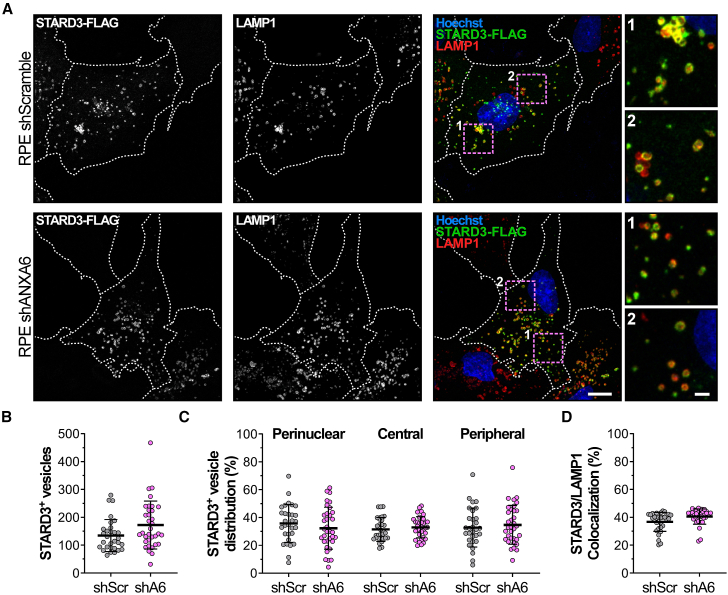
Distribution of LE/Lys vesicles in ANXA6-knockdown RPE cells (A) Representative confocal immunofluorescence images of transfected STARD3-FLAG (green) and LAMP1 (red), and (B–D) quantifications of vesicle number (B), distribution (C), and colocalization (D) of shRNA Scramble and shANXA6 RPE cells (*n* > 50 cells, 2 independent measurements). Scale bars, 10 μm; inset scale bars, 2 μm. Data are represented as mean ± SD. Statistical significance was calculated using Student’s *t* test.

For comparison, RPE-shScramble and RPE-shANXA6 cells also showed a similar staining patterns of LAMP1- and CD63-positive vesicles (Figures 5A and S5A–S5D). However, despite similar numbers of STARD3-positive vesicles in these two RPE cell lines (Figure 5C), no significant repositioning of STARD3-positive LE/Lys to the periphery upon ANXA6 depletion was observed.

### ANXA6 depletion decreases microvillar structures and focal adhesion numbers

Structural support ensures adaptability at the cell surface for appropriate communication with the microenvironment by combining inputs from mechanical forces, signaling events, the cortical ER, a plethora of ABPs, myosins, and other cytoskeletal structures.^48^^,^^49^^,^^50^ As ANXA6 interacts with the actin cytoskeleton and ABPs (e.g., SPTBN1) (Table S3) and ANXA6 depletion caused a dramatic shift toward cytoskeletal-associated proteins in the STARD3 interactome (Tables S4 and S5), we hypothesized morphological differences at the cell surface and, therefore, compared WT and ANXA6ko cells by scanning electron microscopy (SEM).

**Figure 6:**
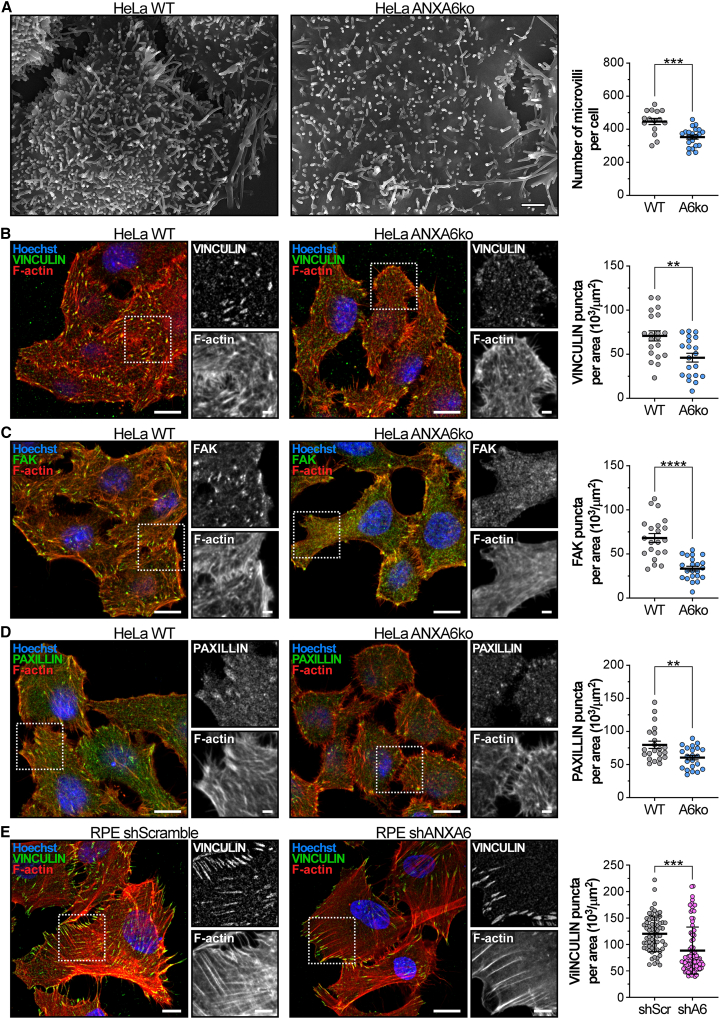
ANXA6 regulates microvilli density and focal adhesion composition (A) Representative scanning electron microscopy (SEM) images of the apical surface morphology and microvilli quantification in WT and ANXA6ko HeLa cells (*n* > 20 from two independent measurements). Scale bar, 2 μm. (B–D) Representative confocal immunofluorescence images of focal adhesions labeled with VINCULIN (B), FAK (C), and PAXILLIN (D) in green, along with F-actin staining (phalloidin) in red in WT and ANXA6ko HeLa cells. (E) Representative confocal immunofluorescence images of VINCULIN in green, along with F-actin staining (phalloidin) in red, in shScramble and shANXA6 RPE cells. Insets highlight focal adhesion puncta, and right panels show quantification of the number of puncta per cell (*n* > 80 cells from 3 independent measurements). Scale bars, 10 μm; inset scale bars, 2 μm. Data are represented as mean ± SEM. Statistical significance was calculated using a two-tailed Mann Whitney test; ∗∗*p* < 0.01, ∗∗∗*p* < 0.001, ∗∗∗∗*p* < 0.0001.

Alike other epithelial cells, HeLa cells are covered with multiple tightly packed microvillar arrays on their apical surface that are supported by bundled actin filaments with their plus end at the tip. Several myosins provide the force to maintain the integrity of microvilli at the membrane.^51^ WT HeLa cells displayed a substantial number of microvilli, while a significant reduction of the number of microvilli upon ANXA6 depletion was evident (Figure 6A).

We then postulated that other structures facilitating cell substrate interactions could also be affected and determined the number of FAs by imaging three FA markers, VINCULIN, focal adhesion kinase (FAK), and PAXILLIN (Figures 6B–6D). These proteins colocalized with the actin cytoskeleton but showed a significant decrease in the number of puncta in ANXA6ko HeLa cells, indicating a reduction in FA numbers. Alike the reduced FA numbers in Anx6-deficient HeLa cells, the quantification of FA puncta using VINCULIN staining also revealed a significant decrease of FA numbers upon ANXA6 depletion in RPE cells (Figure 6E).

These observations did not significantly impact cell spreading compared to controls (Figure S6A). Yet, in line with previous findings,^52^ cell velocity in wound healing assays of ANXA6-depleted HeLa cells was strongly (50%) increased (Figure S6B), indicating that reduced microvilli and FA in cells lacking ANXA6 contribute to cell behavior and their interaction with the environment.

Taken together, ANXA6 depletion causes increased peripheral localization of a subset of STARD3-positive LE/Lys that likely promotes the interaction of STARD3 with cortical cytoskeletal proteins to induce multiple changes in the cell surface organization and function.

## Discussion

Several annexins have recently been associated with the regulation of MCS formation. Here, we demonstrated that ANXA6 depletion modulates MCS formation and numbers in HeLa and RPE cells. Strikingly, this was associated with significant changes in the protein interaction network of the late endosomal cholesterol transporter STARD3 and a redistribution of STARD3-positive LE/Lys vesicles to the cell periphery in HeLa cells. In this location, STARD3 appeared to increasingly interact with the cortical actomyosin cytoskeleton, which coincided with changes in microvillar structures and FA numbers. Importantly, restoration of ANXA6 expression in ANXA6-deficient HeLa cells re-established MCS numbers, indicating that transient changes in ANXA6 expression levels can significantly impact MCS dynamics.

### ANXA6 regulates membrane contact site formation between LE/Lys, ER, and mitochondria

ANXA6 was previously considered to (1) organize membrane domains, (2) create a scaffold for multifactorial signaling complexes, (3) regulate transient membrane-actin interactions during endocytosis, and (4) modulate cholesterol homeostasis.^53^^,^^54^^,^^55^

Recent work from our laboratories also implicated ANXA6 in MCS formation. Using NPC1-mutant CHO cells characterized by cholesterol accumulation in LE/Lys, we demonstrated ANXA6 depletion to enable STARD3-mediated cholesterol transfer from LE/Lys to the ER.^22^ Here, we assessed a potential role for ANXA6 in MCS formation in HeLa cells with functional NPC1. Remarkably, TEM analysis and PLAs revealed that ANXA6 deficiency was associated with a reduced length of membrane contacts between LE/Lys-mitochondria and ER-mitochondria. In line with these results, reduced ER-mitochondria connectivity in ANXA6 knockout mice has been described,^56^ altogether identifying ANXA6 as a regulator of multiple MCS formation.

MCS organization/assembly in cells lacking ANXA6 may be linked to the loss of a physical association and tethering function of ANXA6 in MCSs. ANXA6 has been identified in hepatic mitochondria-associated membranes, a specialized subdomain of the ER that participates in the regulation of intracellular steroid and lipoprotein metabolism-related processes.^57^ Also, the ANXA6 interactome contained several MCS-associated proteins. ANXA6 consists of two core domains, each with four repeats that have disk-like shapes. A flexible linker enables the two ANXA6 disks to be orientated perpendicularly to one another and bind membranes in parallel or antiparallel orientation, which could allow ANXA6 binding two membranes simultaneously.^6^ In addition, the ANXA6 linker region (amino acid [aa] 321–364) contains 3 putative interaction domains relevant in the context of MCSs. First, a KGD motif (aa 354–356) with potential for binding integrins or C2 domains can mediate Ca^2+^-inducible membrane targeting of proteins.^58^^,^^59^ Within this motif, phosphorylation of a Thr (T) residue (aa 356) triggers conformational changes that could provide ANXA6 the flexibility required to initiate contacts between membranes.^60^ Second, a WE motif (aa 343–344) resembles the hydrophobic and acidic membrane attachment motif that facilitates lysosomal targeting of proteins.^61^ As part of this motif, the W343 residue provides the cholesterol-binding properties of ANXA6.^62^ Third, an FFAT-like domain (aa 331–337) with a highly conserved acidic Asp (D) residue and a double Phe (FF) followed by Glu-Ala (EA), according to an algorithm developed by Levine and colleagues,^63^ has a high likelihood to associate with MCS (score = 4.0). In addition, another FFAT-like sequence (FFNT; aa 54–70) within the first annexin repeat also displays a high score to associate with MCS (score = 3.5),^13^ and a recent update of the VAPome located two motifs in the ANXA6 repeats 1 and 2 with similar elevated predictability scores (3.5 each).^15^

The scaffold properties of ANXA6 could also participate in the assembly or stabilization of MCS involving BCAP31 in the ER or MFN1 and TSPO in mitochondria. Conformational changes due to Ca^2+^ or T356 phosphorylation in the linker region could allow ANXA6-dependent dynamic changes in MCS formation in response to extracellular signals and environmental changes.^60^ One can speculate that ANXA6 deficiency may cause variations within the space between membrane neighbors with consequences for organelle connectivity.

### STARD3 is a multifunctional tether that associates LE/Lys with the cortical actin cytoskeleton in ANXA6-depleted cells

ANXA6 is an abundant protein in HeLa cells.^64^ Although synthesized in the cytosol, the continuous Ca^2+^ exchanges between organelles and the cytosol induce preferential targeting of ANXA6 to associate with membranes, such as endosomes and the PM, and feasibly other organelles.^8^^,^^56^ ANXA6 depletion reduced membrane contacts and impacted the protein interaction networks of the LE/Lys-resident protein STARD3, a well-known tether in MCS between LE/Lys and ER and possibly between LE/Lys and mitochondria.^36^^,^^37^^,^^38^^,^^39^^,^^65^^,^^66^^,^^67^ In particular, STARD3 interacts with the ER-resident proteins VAPA/B and MOSPD2, through its phospho-FFAT motif.^19^^,^^68^^,^^69^

Notably, ANXA6 deficiency caused a substantial reduction in the number of MCS-associated proteins within the STARD3 interactome. For instance, ANXA6 depletion was linked with a loss of STARD3 interactors located in the ER (e.g., VAPB, MOSPD2, and BCAP31) and in mitochondria (e.g., ATAD3A and MFN1). A significant diminution of STARD3-TSPO interaction was also observed. Hence, ANXA6 may assist with MCS formation, while its depletion induces membrane reorganizations that entail STARD3-related inter-organelle communication. Altogether, ANXA6 is a key player that coordinates the formation of STARD3-containing MCSs.

Unexpectedly, the STARD3 interactome from WT and ANXA6-deficient HeLa cells contained a large number of cytoskeletal-associated proteins, including components of both microtubule and actin filaments. This indicates that STARD3 communicates with the actin cytoskeleton and associated myosin motors that modulate the transport of LE/Lys. This LE/Lys vesicle movement occurs through several mechanisms, possibly as part of lysosomal exocytosis, toward the actomyosin cortical cytoskeleton underlying the PM. In WT cells, the STARD3 interactome contained several tubulins and dynein, as well as ABPs such as spectrin (SPTBN1), filamin A (FLNA), and Ezrin, while myosins were lacking. In contrast, in ANXA6-deficient cells, STARD3 also interacted with tubulins, SPTBN1, dynein, and kinesin KIF5B, yet there was a notable enrichment of STARD3 interaction with non-muscular myosins (conventional and non-conventional) as well as other ABPs including gelsolin or actin itself.

Intriguingly, these findings support earlier work reporting a link between STARD3 and the actin cytoskeleton.^70^ STARD3 depletion caused a disorganization of the actin cytoskeleton similar to the disruption of actin polymerization observed when employing cytochalasin D. In these studies, STARD3 depletion strongly reduced the proportion of STARD3-positive LE that were in contact with phalloidin-stained actin filaments.^70^

14-3-3 proteins are also believed to connect STARD3 to the actin cytoskeleton. For instance, STARD3 interaction with 14-3-3 eta serves as a bridge between STARD3 and actin filaments, possibly facilitating lysosomal positioning in coordination with the ER.^71^ Also, 14-3-3 theta/tau participate in STARD3 trafficking to lysosomes.^72^ Although the specific functions of these interactions remain largely unexplored, we identified 14-3-3 eta, theta, and epsilon proteins as STARD3 interactors in ANXA6ko, but not WT cells. In contrast, the STARD3 interactome of WT cells contained 14-3-3 isoforms theta/tau, zeta, gamma, and beta. Hence, these alterations in STARD3 and 14-3-3 interactions could reflect an ANXA6-dependent change in the positioning and trafficking of STARD3 along actin filaments toward the cell periphery.

Most striking was the robust presence of myosins in the STARD3 interactome from ANXA6ko cells. Among those, we identified and validated MYH9 as an interactor of STARD3. Notably, depletion of ANXA6 led to a marked repositioning of a subset of STARD3-positive LE/Lys, which correlated with changes in proteins involved in membrane trafficking pathways, including LE/Lys proteins. Besides MYH9, other hits in the ANXA6- or STARD3-based proximity-dependent biotinylation assays are linked to LE/Lys positioning, such as ARL8B, RUFY3, SYTL4, RAB3, MYO1C, and MYO5A.^43^^,^^44^^,^^45^^,^^46^^,^^47^

### The STARD3/TSPO tethering complex between endolysosomes and mitochondria

STARD3 interactomes from WT and ANXA6ko cells both contained a considerable number of mitochondrial-associated proteins, such as FKBP8, TSPO, ACSL1, OPA1, SAMM50, VDAC3, and several SLC25A channel proteins from the outer and inner mitochondrial membrane (e.g., MICOS complex).

These observations reinforce the hypothesis that STARD3 functions as a lysosomal tether with mitochondria, a critical inter-organelle contact for the regulation of metabolism. Moreover, we identified an interaction between STARD3 and TSPO, a highly conserved protein with roles in cholesterol transport and steroidogenesis and several other mitochondrial and cellular functions.^34^^,^^73^ TSPO contains a specific consensus motif for cholesterol binding and has been implicated in mitochondrial lipid metabolism.^74^^,^^75^ The interaction between STARD3 and TSPO introduces a new tethering pair for MCS between LE/Lys and mitochondria. Interestingly, TSPO also interacts with NPC1,^76^ while other studies reported STARD3 to mediate cholesterol transfer from LE/Lys to mitochondria in NPC1-deficient cells, suggesting multiple lipid exchange routes between these two organelles. This identified STARD3-TSPO interaction may help explain such phenotypes in the absence of NPC1.

Although our data support a working model in which ANXA6 modulates STARD3-TSPO tethering and actomyosin dynamics, these conclusions are primarily based on correlative interactome analyses and rescue experiments in WT cells. Consequently, the precise molecular sequence of events and the specific domains mediating these interactions, remain to be elucidated in future studies.

Annexins have roles in the proper connectivity between LE/Lys, ER, and mitochondria, which is essential for cellular homeostasis, and its dysfunction in these inter-organelle communications is associated with pathophysiological outcomes. This includes the lack of MCS formation between LE/Lys and the ER during development of neurodegenerative diseases such as NPC, contributing to the accumulation of lipids (cholesterol and sphingolipids).^5^ Also, incorrect wiring of MCS can lead to the missorting of ligands to undesired destinations, as shown for the increased MCS formation and associated elevated cholesterol transfer from LE/Lys to mitochondria in NPC1-mutant hepatocytes, causing oxidative stress and mitochondrial dysfunction.^77^^,^^78^ The recent discovery of dysregulated MCS formation in disease settings has stimulated research to uncover potential therapeutical targets that could overcome pathophysiological consequences due to improper MCS formation. Therefore, the understanding how cells exploit MCS formation to modulate ion or lipid metabolism and transport, signaling, or organelle positioning becomes essential in health and disease.

### Limitations of the study

Although proximity-based proteomic approaches provide a broad overview of the molecular environment, they may present inherent detection biases, including problems due to overexpression of the bait, an underrepresentation of low-abundance proteins, or highly transient interactions. In addition, the experiments shown in this manuscript were performed in HeLa and RPE cells, cellular models that may not fully recapitulate physiological contexts. Therefore, validation in more relevant cellular or *in vivo* models will be necessary to assess the generalizability of these findings.

## Resource availability

### Lead contact

Further information and requests for resources and reagents should be directed to the lead contact, Carles Rentero (carles.rentero@ub.edu).

### Materials availability

Plasmids generated in this study will be made available upon request.

### Data and code availability

• The mass spectrometry proteomic data produced in this study have been deposited to the ProteomeXchange Consortium via the PRIDE partner repository with the dataset identifier PXD078364.
• The code for the ImageJ macro analysis for vesicle distribution and colocalization is available at https://doi.org/10.5281/zenodo.16538283.
• Data reported in this paper are available from the lead contact upon request.
• Any additional information required to reanalyze the data reported in this paper is available from the lead contact upon request.

## Acknowledgments

This study was supported by a Ministerio de Ciencia e Innovación (Spain) research grant PID2020-115910RB-I00 to C.E. and C.R.; PID2022-138728OB-I00 to N.A. and A.L., and grant 2021SGR00284 from the Agència de Gestió d’Ajuts Universitaris i de Recerca (AGAUR) (Spain). T.G. is supported by the University of Sydney, Australia (227638 and 227919) and the Ara Parseghian Research Foundation (G217137). Y.L. is supported by the Chinese Research Council. A.R.A. and S.Z. are supported by Agencia Nacional de Investigación y Desarrollo (ANID, Chile): Fondecyt grants 1241503 and 1230337. P.A.G. is supported by Master Plus Collaboration Scholarship (Universitat de Barcelona). We thank the staff of the Centers Científics i Tecnològics, Universitat de Barcelona (CCiTUB), Electron Microscopy and Advance Optical Microscopy Units, for their assistance. The proteomics analyses were performed in the CRG/UPF Proteomics Unit that is part of the Spanish National Infrastructure for Omics Technologies (ICTS OmicsTech).

## Author contributions

M.B.-E. conducted most of the biochemical and imaging experiments, formal analysis, quantifications, and data analyses; Y.L. contributed to some of the biochemical and imaging experiments; E.P. helped with the EM methodology; J.M.E. performed the mass spectrometry experiments; G.M. and M.C. contributed to protocols, design, and confocal image analysis; P.A.G. contributed to confocal image analysis; M.K.L.N. contributed to some of the biochemical experiments; A.R.A., S.Z., and N.A. conceptualized and helped in data analysis and discussions; A.L. conceptualized and helped in discussions and design of experiments; F.T. supervised the project and the methodology and contributed to data analysis and discussions; C.E. performed all EM experiments, drafted the original manuscript, and supervised the project; T.G. conceptualized the study and performed data analysis and manuscript writing and editing; C.R. supervised the project and the methodology, contributed to formal analysis, and designed the DNA construct protocols. All authors reviewed and approved the final version of the manuscript.

## Declaration of interests

The authors declare no competing interests.

## STAR★Methods

### Key resources table

**Table:** 

REAGENT or RESOURCE	SOURCE	IDENTIFIER
**Antibodies**		

Rabbit polyclonal anti-ANXA6	Self-produced in the laboratory^79^^,^^80^	N/A
Mouse monoclonal anti-β-ACTIN (AC-15)	Sigma Aldrich	Cat# A5441; RRID: AB_476744
Mouse monoclonal anti-FAK (77/FAK)	BD Transduction	Cat# 610088; RRID: AB_397495
Mouse polyclonal anti-CD63	Gift from Dr. Francisco Sánchez-Madrid	N/A
Mouse monoclonal anti-FLAG (M2)	Sigma Aldrich	Cat# F1804; RRID: AB_262044
Rabbit monoclonal anti-HA (C29F4)	Cell Signaling	Cat# 3724; RRID: AB_1549585
Rabbit monoclonal anti-LAMP1 XP (D2D11)	Cell Signaling	Cat# 9091; RRID: AB_2687579
Mouse monoclonal anti-LAMP1 (H4A3)	Milipore	Cat# MABC1108; RRID: AB_2923037
Rabbit polyclonal anti-MYH9	BioLegend	Cat# 909801; RRID: AB_2565100
Mouse monoclonal anti-PAXILLIN (177/Paxillin)	BD Transduction	Cat# 610568; RRID: AB_397917
Rabbit monoclonal anti-TOMM20 (EPR15581)	Abcam	Cat# ab186734; RRID: AB_2716623
Mouse monoclonal anti-TOMM20 (1D6F5)	Proteintech	Cat# 66777-1-Ig; RRID: AB_2882123
Rabbit monoclonal anti-TSPO (SA90-03)	Invitrogen	Cat# MA5-31966; RRID: AB_2809260
Rabbit polycolona anti-VAPB	Atlas Antibodies	Cat# HPA013144; RRID: AB_1858717
Mouse monoclonal anti-VINCULIN (VIN-11-5)	Sigma Aldrich	Cat# SAB4200729; RRID: AB_2877646

**Secondary Antibodies**		

Alexa Fluor 488 goat anti-mouse IgG	ThermoFisher	Cat# A28175; RRID: AB_2536161
Alexa Fluor 555 goat anti-mouse IgG	ThermoFisher	Cat# A28180; RRID: AB_2536164
Alexa Fluor 488 goat anti-rabbit IgG	ThermoFisher	Cat# A32731; RRID: AB_2633280
Alexa Fluor 555 goat anti-rabbit IgG	ThermoFisher	Cat# A21428; RRID: AB_2535849
HRP goat anti-mouse IgG	BioRad	Cat# 1706516; RRID: AB_2921252
HRP goat anti-rabbit IgG	BioRad	Cat# 1706515; RRID: AB_11125142
Duolink® *In Situ* PLA® Probe Anti-Mouse PLUS	Merck Life Science SLU	Cat# DUO92001-100RXN; RRID: AB_2810939
Duolink® *In Situ* PLA® Probe Anti-Rabbit MINUS	Merck Life Science SLU	Cat# DUO92005-100RXN; RRID: AB_2810942

**Other proteins**		

Alexa Fluor 488 Streptavidin	ThermoFisher	S11223
HRP Streptavidin	ThermoFisher	N100

**Recombinant DNA**		

pSpCas9(BB)-2A-Puro v2 px495	Addgene	#48139
pEGFP-ANXA6	Cubells et al.^54^ and Vila de Muga et al.^81^	N/A
pCMV-STARD3-EGFP	From Prof. Elina Ikonen	N/A
pCMV-STARD3-FLAG	This paper	N/A
pCMV-STARD3-ΔSTART-FLAG	This paper	N/A
pCMV-miniTurboID	Addgene	#209637
pSF3-UltraID	Addgene	#172878
pLJC5-TMEM192-3xHA	Addgene	#102930
pCMV-TMEM192-miniTurboID	This paper	N/A
pCMV-STARD3-miniTurboID	This paper	N/A
pCMV-ANXA6-UltraID	This paper	N/A
pLKO.1-TRC cloning vector	Addgene	#10787
pLKO.1-Scramble	Addgene	#1864
pLKO.1-human ANXA6	This paper	

**Oligonucleotides**		

Human *TBP* Fw 5′- CGCAAGGGTTTCTGGTTTGC -3′	This paper	N/A
Human *TBP* Rv 5′- AATAGGCTGTGGGGTCAGTC -3′	This paper	N/A
Human *ANXA6* Fw 5′- CTGCGTCCTCGAGTCCCTG -3′	This paper	N/A
Human *ANXA6* Rv 5′- TGTTGCTACCGTAAGGTGATT -3′	This paper	N/A
Human ANXA6 sgRNA Fw 5′- caccGCAGAGCTACAAGTCCCTCTA -3′	This paper	N/A
Human ANXA6 sgRNA Rv 5′- aaacTAGAGGGACTTGTAGCTCTGC -3′	This paper	N/A
Human *ANXA6* shRNA Fw 5′- ccggCGGGCACTTCTGCCA AGAAATctcgagtATTTCTTGGCA GAAGTGCCCGtttttg -3′	This paper	N/A
Human *ANXA6* shRNA Rv 5′- aattcaaaaaCGGGCACT TCTGCCAAGAAATactcga gATTTCTTGGCAGAAGTGCCCG -3′	This paper	N/A

**Chemicals**		

U18666A	Sigma Aldrich	662015
Phalloidin, rhodamine conjugated	Sigma Aldrich	R415
Hoechst	ThermoFisher	33342

**Software and Algorithms**		

ImageJ	^82^	N/A
GraphPad Prism 10	http://www.graphpad.com	N/A
CRISPR design	https://benchling.com	N/A
ImageJ macros for vesicle distribution and colocalization analysis	This paper (https://doi.org/10.5281/zenodo.16538283)	N/A

**Data availability**		

Proteomic data produced in this study is available via ProteomeXchange (PRIDE)	https://www.ebi.ac.uk/pride/	PXD078364

### Experimental model and study participant details

#### Cell culture and transfections

HeLa (kindly provided by Prof. G. Scita, IFOM, Italy), HEK293T (ATCC, CRL-3216) and hTERT RPE-1 (ATCC, CRL-4000) cell lines were grown in Dulbecco’s Modified Eagle’s Medium (DMEM, high glucose; Diagnovum D009-500) supplemented with 10% fetal bovine serum (Diagnovum, D016-500), 10 U/mL penicillin and 10 μg/mL streptomycin (Diagnovum, D910-100), 4 mM L-glutamine (Sigma Aldrich, 49419), 1 mM sodium pyruvate (Sigma Aldrich, P-5280) and non-essential amino acids (100X, Diagnovum, D603-100) at 37 °C in a humidified atmosphere with 5% CO_2_. These cell lines were not authenticated. Cells were regularly tested for mycoplasma contamination using a PCR-based detection assay.

For transient transfections, cells were incubated for 16 h with GenJet Plus Reagent (SigmaGen Laboratories, SL100499) following manufacturer’s instructions.

### Method details

#### Generation of HeLa-ANXA6ko cells using the CRISPR/Cas9 system

For *ANXA6* gene depletion using CRISPR/Cas9 editing technology, guide RNAs targeting human *ANXA6* were designed as described.^83^ HeLa cells were transfected with pSpCas9(BB)-2A-Puro v2 (Addgene #48139) carrying gRNAs against human *ANXA6*. 24 h after transfection, cells were selected for 48 h in puromycin (2 μg/mL, Sigma Aldrich, P9620). Clones were isolated by dilution and single clones were screened for *ANXA6* gene knockout by qPCR and western blotting and sequencing.

#### shRNA-mediated inhibition of annexin A6 expression

For human *ANXA6* mRNA levels reduction in RPE cells, shRNA primers were designed and cloned in pLKO.1-TRC cloning vector (Addgene # 10787). Scramble shRNA pLKO.1 vector was used as control (Addgene #1864). For 3rd generation lentiviral particle production, pMDLg/pRRE, pMD2.G and pRSV-Rev were cotransfected with pLKO.1-shAnxA6 in HEK293/T using GenJet Plus. Media was replaced with normal medium 8 h after transfection. Virus-containing supernatants (VCS) were harvested and filtered (0.45 μm) on day 2 and 3 after transfection and stored at 4 °C until use. For lentiviral transduction, RPE cells were plated into 6-well plates and infected with 0.33 mL VCS/mL fresh media for 24 h and selected with 4 μg/mL of puromycin (Sigma-Aldrich, P9620-10 ML) for other 48 h.

#### RNA extraction and quantitative real-time PCR

Total RNA was extracted with the EZ-10 RNA Mini-prep kit (Bio Basic, BS88136) in accordance with the manufacturer’s protocol. 1 μg RNA was reverse-transcribed using the High-Capacity cDNA Reverse Transcription Kit (Applied Biosystems, 4368814). In a final volume of 20 μL real-time PCR Brilliant SYBRGreen GoTaq qPCR Master Mix (Promega, A6002), 10 μL cDNA (diluted 1:20) was used as a template for PCR analysis using the LightCycler system (Roche Diagnostics), together with specific primers (see Table S1). Standard PCR amplification protocol was performed (10 min at 95 °C; 45 cycles of 30 s at 95 °C, 15 s at 60 °C and 30 s at 72 °C; and 10 s at 95 °C and 60 s at 65 °C) according to manufacturer’s instructions. Values were normalized to the Tata-box binding protein (TBP) gene in each sample.

#### Proximity-dependent biotinylation

For the proximity-dependent biotinylation (PDB), cells were seeded in 100 mm Petri dishes and transfected with ANXA6-ultraID, ultraID, STARD3-miniTurboID or TMEM192-miniTurboID.^29^ Twenty-four hours after transfection, cells were incubated for 30 min with complete media supplemented with 50 μM biotin (Sigma Aldrich, B4501). Cells were then washed in PBS and lysed in lysis buffer (8 M urea, 1 mM dithiothreitol (DTT) and 50 mM Tris-HCl, pH 7.4) supplemented with protease/phosphatase inhibitors cocktail. Lysates were sonicated for 30 s on ice and Triton X-100 was added to 1% final concentration. Lysates were then incubated with streptavidin-conjugated C1 Dynabeads MyOne (ThermoFisher, 65001) for 1 h at 4 °C. Streptavidin beads were washed (5 min at room temperature) twice with wash buffer 1 (2% SDS), twice with wash buffer 2 (0.1% sodium-deoxycholate, 1% Triton X-100, 500 mM NaCl, 1 mM EDTA and 50 mM HEPES, pH = 7.4), twice with wash buffer 3 (250 mM lithium chloride, 1 mM EDTA, 0.5% Igepal CA-630, 0.1% sodium-deoxycholate and 10 mM Tris-HCl, pH = 8.0), and finally 3 times with wash buffer 4 (50 mM ammonium bicarbonate).^29^

After biotinylated protein enrichment, STARD3-miniTurboID and TMEM192-miniTurboID samples were resolved in 10% acrylamide sodium dodecyl sulfate polyacrylamide gel electrophoresis (SDS-PAGE) gel. The gel was visualized with silver staining and each lane was subsequently excised into 16 gel slices. Gel slices were washed with 25 mM ammonium bicarbonate and dehydrated with 100% acetonitrile; this was repeated three times. Gel slices were then reduced with 5 mM DTT in 25 mM ammonium bicarbonate at 65 °C for 1 h. Cysteines were blocked with 55 mM iodoacetamide in 25 mM ammonium bicarbonate (pH 8), and samples were incubated overnight with 5 μL of sequencing grade trypsin 12.5 μg/mL solution in 50 mM ammonium bicarbonate (pH 8.0) at 37 °C. Peptides were extracted with 5 μL of 0.1% trifluoroacetic acid in water.

After biotinylated protein enrichment, ANXA6-ultraID and ultraID samples were digested on the bead overnight with 5 μL of sequencing grade trypsin 12.5 μg/mL solution in 50 mM ammonium bicarbonate (pH 8.0) at 37 °C. Samples were analyzed using a Orbitrap Fusion Lumos mass spectrometer (Thermo Fisher Scientific) coupled to an EASY-nLC 1200 (Thermo Fisher Scientific (Proxeon)). Peptides were loaded directly onto the analytical column and were separated by reversed-phase chromatography using a 50-cm column with an inner diameter of 75 μm, packed with 2 μm C18 particles (Thermo Fisher Scientific).

Protein identification, label-free quantification and normalization were performed using Proteome Discoverer 3.1 (ThermoFisher Scientific), searching against the appropriate Uniprot reference proteome with carbamidomethylation (C) as a fixed modification and oxidation (M), phosphorylation (S, T, Y), biotinylation (K) and N-terminal acetylation as variable modifications. Normalized protein abundances were further analyzed using Perseus (Max Plank Institute). Proteins identified with only a single peptide-spectrum match (PSM) were excluded from further analysis. Missing values in the data matrix were imputed based on a normal distribution. To calculate the False Discovery Rate (*p*-value), the mean protein abundances from biological triplicates of each condition were compared against each control (TMEM192-miniTurboID or UltraID) using a two-tailed Student’s *t* test, with a significance threshold set at *p* < 0.05. The *p*-value and abundance data were -log_10_ and log_2_ transformed respectively and visualized as a Volcano plot generated using RStudio (Posit Software). To consider a specific protein as interactor we selected *p* < 0.05 and fold-change >2 as thresholds.

Prior to LC-MS/MS analysis, quality and expression controls were performed. Biotin ligase-bait chimeric protein expression and location was assessed by immunofluorescence using streptavidin-Alexa Fluor 488 (ThermoFisher, S11223), by immunoblotting using streptavidin-HRP (ThermoFisher, N100) and by silver staining (see Figure S3).

#### Immunoblotting

Cells were lysed in lysis buffer (50 mM HEPES, pH 7.4, 1% Triton X-100, 10% glycerol) supplemented with protease/phosphatase inhibitors cocktail (10 μg/mL aprotinin; Sigma Aldrich, A1153-25 MG), 10 μg/mL leupeptin (Sigma Aldrich, L2884-25 MG), 1 mM Na_3_VO_4_ (Sigma Aldrich, S6508), 10 mM NaF (Sigma Aldrich, S7920) and 1 mM phenylmethylsulfonyl fluoride (PMSF; Sigma Aldrich, P7626). Lysates were centrifuged at 14,000g for 15 min at 4 °C to obtain the post-nuclear supernatants (PNS). Protein concentration in cell lysates was determined using the Bradford assay following standard protocols (Bio-Rad Protein Assay Dye Reagent Concentrate, 500-0006). The PNS were boiled in 1 × Laemmli buffer, resolved on SDS-PAGE and transferred to Immobilon-P (Millipore, IPVH00010) membranes. Membranes were blocked in 5% non-fat milk, incubated overnight in primary antibodies, washed in TBST, incubated with HRP-conjugated secondary antibodies (Bio-Rad, see STAR Methods) and developed using enhanced chemiluminescence EZ-ECL (Enhanced Chemiluminescence, NZYTech, MB40101). ImageJ software was used for quantitative analysis of Western blot bands.

#### Immunoprecipitation

Cells were lysed using immunoprecipitation buffer (150 mM NaCl, 10 mM HEPES, 2 mM EDTA, 1% Triton X-100, 1.5 mM MgCl_2_, 10% glycerol, and protease Inhibitor cocktail) and centrifuged at 14,000g for 15 min at 4 °C. The PNS were incubated with magnetic beads (Dynabeads 10003D, Thermo Fisher) conjugated to 2.5 μg rabbit anti-TSPO antibody or 2.5 μg mouse anti-FLAG antibody at 4 °C overnight. After magnetisation, the resulting pellet was washed and mixed with 35 μL 1× SDS-PAGE sample buffer and heated for 10 min at 95 °C followed by magnetisation and collection of the supernatant for further immunoblotting analyses.

#### Immunofluorescence

Cells grown on coverslips were fixed with 4% paraformaldehyde (PFA, Electron Microscopy Sciences, 15710) for 15 min at room temperature (RT), washed with PBS, permeabilized with 0.1% saponin for 10 min and blocked with 1% bovine serum albumin (BSA) for 5 min. Coverslips were incubated with primary antibody diluted in 0.02% saponin, 0.1% BSA in PBS for 1 h at RT, washed intensively and then incubated with the appropriate AlexaFluor-conjugated secondary antibodies for 45 min at RT. After staining, coverslips were mounted in Mowiol (Sigma Aldrich, 81381). Samples were visualized using either a Leica AF6000 epifluorescence inverted microscope equipped with an HCX PLAN APO 63× oil immersion objective lens or the Zeiss Laser Scanning Confocal Microscope 880 equipped with a blue diode (405 nm), Argon (458/476/488/496/514 nm), diode pumped solid state (561 nm), HeNe (594/633 nm) lasers and APO 63× oil immersion objective lens.

#### Electron microscopy

For Transmission Electron Microscopy (TEM) or Scanning Electron Microscopy (SEM) preparation and analysis, cells were grown on coverslips and first washed with 0.1 M phosphate buffer (PB, pH = 7.4) to remove the excess of culture medium. Cells were then fixed with freshly made 3% glutaraldehyde solution in 0.1 M PB for 1 h at RT. Thereafter, the fixative was removed, and samples were maintained at 4 °C in a fresh fixative solution until processing.

For TEM, the post-fixation procedure was conducted using 1% OsO_4_ for a period of 90 min at 4 °C. Following this, samples were dehydrated in increasing ethanol solutions. Coverslips with cells were placed on top of BEEM capsules filled with Spurr resin (Electron Microscopy Sciences). The polymerization of the resin was carried out at a temperature of 60 °C in a stove for a period of three days. At this point, the glass was removed by thermal contrast switch leaving the cell monolayer remaining at the top of the polymerized block.^23^ The sectioning process was conducted using a Leica ultramicrotome EM UC7 (Leica Microsystems). Ultra-thin sections (60–70 nm) were mounted on cooper grids and stained with 2% Uranyl-less solution for 10 min and with a lead-staining solution for 5 min. The sections were then observed using a JEOL JEM-1010 transmission electron microscope (JEOL Ltd) coupled with an Orius SC1000 CCD camera (model 832; Gatan Inc) at the Electron Microscopy Unit, Scientific and Technological Centers, University of Barcelona.

For SEM, the post-fixation procedure was conducted using 1% OsO_4_ in the same phosphate buffer, dehydrated in graded alcohol and processed for critical point drying using Emitech K850. Samples were covered with a carbon thin film in order to improve their electrical conductivity. The samples were observed with a Jeol JSM-7001F (Jeol) operated at 15 kV at the Electron Microscopy Unit, Scientific and Technological Centers, University of Barcelona.

#### Proximity ligation assay

To assess the number of MCS between two organelles, proximity ligation assays (PLA Duolink, Sigma Aldrich) were performed following manufacturer’s instructions. Primary antibodies were tested to ascertain their specificity and a control condition using only the secondary antibodies was performed (see Figure S2). For lysosome-mitochondria PLA: anti-LAMP1 (Milipore, MABC1108) and anti-TOMM20 (Abcam, ab186734); for lysosome-ER PLA: anti-LAMP1 (Milipore, MABC1108) and anti-VAPB (Atlas Antibodies, HPA013144); and for ER-mitochondria PLA: anti-VAPB (Atlas Antibodies, HPA013144) and anti-TOMM20 (Proteintech, 66777-1-Ig).

#### Wound healing assay

Cells were seeded in μIbidi 8-well plates (Ibidi, 80826) and grown to full confluence in complete medium. Once a uniform monolayer was formed, a linear scratch (‘wound’) was created in the cell layer using a sterile 200 μL pipette tip. Detached cells and debris were removed and cells were treated with 5-fluorouridine (Sigma Aldrich, F5130) to inhibit the cell cycle. Images of the wound area were captured every 0.5 h using the EPI TIRF Nikon microscope. Wound closure was quantified using the ImageJ plug-in described previously.^84^ Migration velocity was expressed as μm/h based on the reduction in wound width over time.

#### Spreading assay

Cells were seeded on coverslips at low confluency and after 2 h, cells were fixed in 4% PFA for 15 min at RT. Cells were permeabilized, blocked and stained for F-actin following the standard immunofluorescence protocol (see above). Images were acquired using the Zeiss LSM880 confocal microscope and analyzed using ImageJ.

### Quantification and statistical analysis

#### Image analysis, vesicle distribution and colocalization assessment

Image analysis was performed using NIH ImageJ software.^82^ When comparing different treatments or cell lines, images were acquired and analyzed systematically using identical microscope settings.

To analyze endolysosome vesicle distribution in cells, a semi-automated ImageJ macro was designed and used to quantify their number, size, fluorescence intensity, and cellular distribution. Specifically, fluorescence microscopy images were locally measured, and after a manual selection of cells, vesicles were identified using ImageJ’s plug-in StarDist^81^ (code available at https://doi.org/10.5281/zenodo.16538283). When vesicles were segmented, the number, size, and fluorescence intensity were calculated from raw images. Relative positioning of cellular vesicles was analyzed by dividing the distance from the nuclei geometric center to the plasma membrane in three zones (perinuclear, central and peripheral). With the vesicle segmentation, an extension of the macro was developed to compare the masks of two different channels of vesicles and stablish the total and zonal colocalization of the labeling. The selected threshold for a colocalization was set at 0.1 μm^2^ of overlap between both masks.

The specific parameters required for vesicle detection were optimized for the Zeiss LSM880 Confocal microscopy using a 63× oil immersion objective and a field of view of 2048x2048 pixels.

#### Statistical analysis

Statistical analysis was performed using GraphPad Prism 10 software (GraphPad Software). Unless specified otherwise, results are presented as the mean ± standard error of the mean (SEM) from at least three biological replicates; technical replicates were averaged within each biological experiment and not used for statistical inference. Prior to applying parametric tests, normality of the data distribution was assessed using the Shapiro-Wilk test.

For comparisons between two groups, an unpaired Student’s *t* test was used if the data followed a normal distribution. In cases where normality was not met, a non-parametric Mann-Whitney U test was applied for unpaired data, and a Wilcoxon test for paired data. Differences were considered statistically significant at a *p*-value <0.05. ∗*p* < 0.05, ∗∗*p* < 0.01, ∗∗∗*p* < 0.001, ∗∗∗∗*p* < 0.0001.
