# Supplementary material for: Annexin A6 controls multi-organelle contact site formation and endolysosomal positioning, and remodels the STARD3 interactome
Source: iScience. 2026 Jun 15;29(7):116387. doi: 10.1016/j.isci.2026.116387 (PMC13285654; doi:10.1016/j.isci.2026.116387)
Supplement: Document S1. Figures S1–S6 and Tables S1–S5 [file mmc1.pdf]

iScience, Volume *29*

Supplemental information

Annexin A6 controls multi-organelle contact site formation and endolysosomal positioning, and remodels the STARD3 interactome

Marc Bernaus-Esqué, Yangjing Liu, Eva Prats, Josep M. Estanyol, Gemma Martin, Maria Calvo, Panagiota Areti Gigourtsi, Mai Khanh Linh Nguyen, Alejandra R. Álvarez, Silvana Zanlungo, Neus Agell, Albert Lu, Francesc Tebar, Carlos Enrich, Thomas Grewal, and Carles Rentero

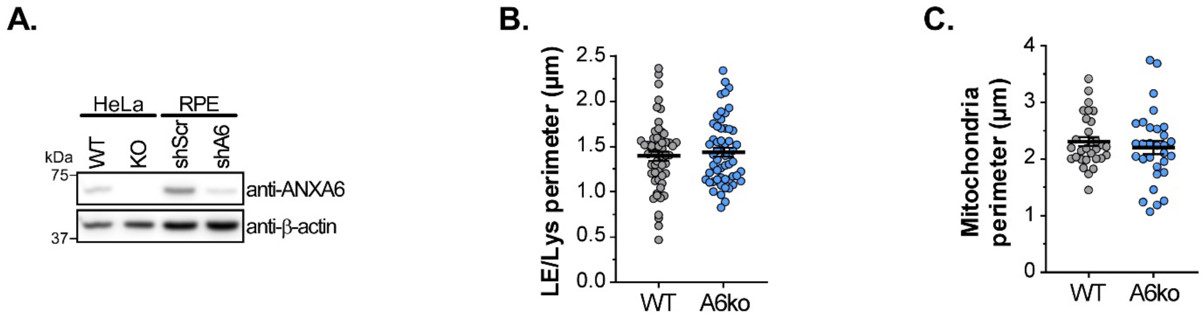

Figure S1. Characterization of ANXA6 depleted HeLa cells. (A) Western blot analysis of ANXA6 protein expression in WT and ANXA6ko HeLa cells, and shRNA Scramble and ANXA6 in pLKO.1 infected RPE cells. (B and C) ANXA6 deletion does not alter MVB/LE/Lys and mitochondria perimeter. TEM quantification of the MVB/LE/Lys (A) and mitochondria (B) perimeters (n > 30 cells, from 3 independent measurements). Data is represented as mean ± SEM. Statistical significance was calculated using a two-tailed Mann Whitney test.

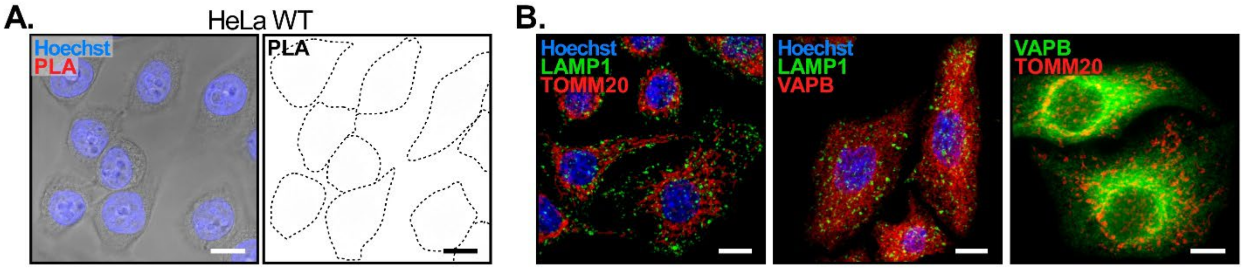

Figure S2. Proximity ligation assay (PLA) antibody control in human cells. (A) Control of the PLA secondary antibodies. (B) Representative confocal immunofluorescence images of the primary antibodies used for the PLA assays. Scale bar = 10 µm.

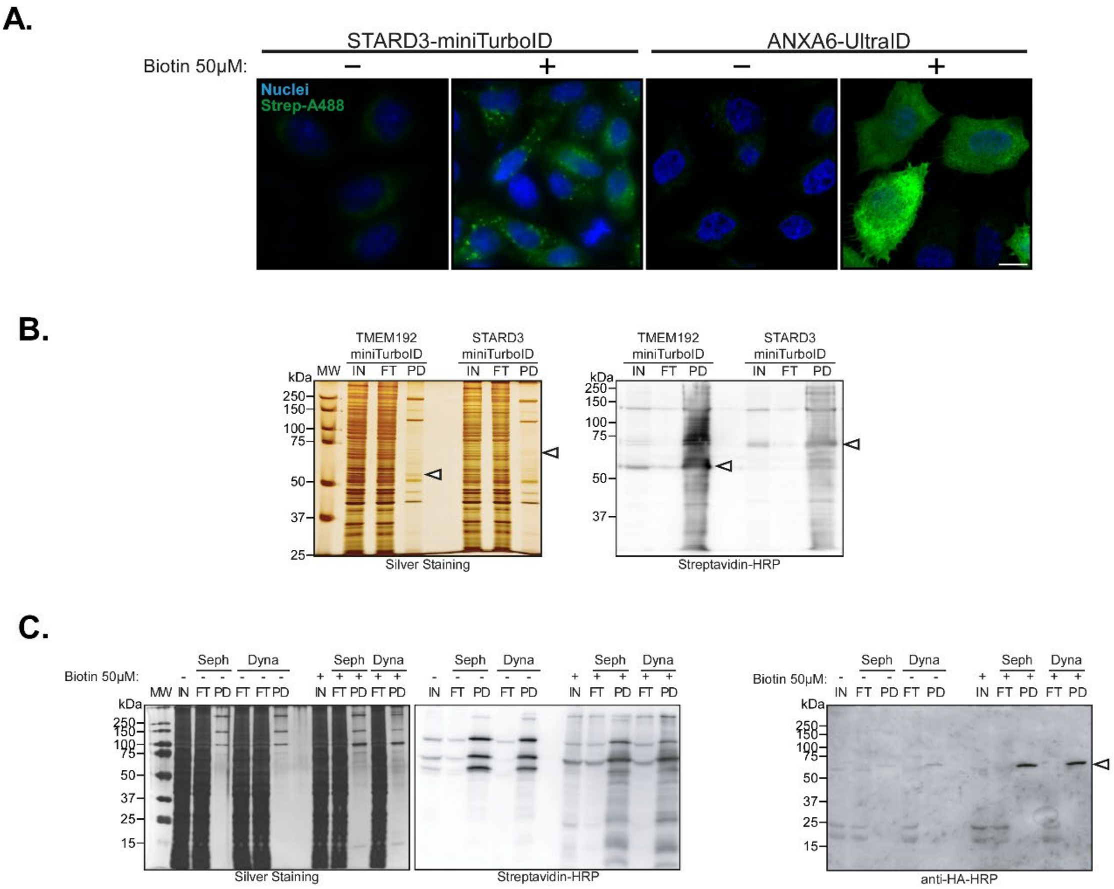

Figure S3. Validation of proximity labelling with miniTurboID and UltraID constructs. (A) Representative widefield images showing biotinylation in cells expressing STARD3-miniTurboID or ANXA6-UltraID. Scale bar = 10 µm. (B, C) Silver staining and streptavidin/HA-HRP blots confirming biotinylated protein enrichment in pull-downs.

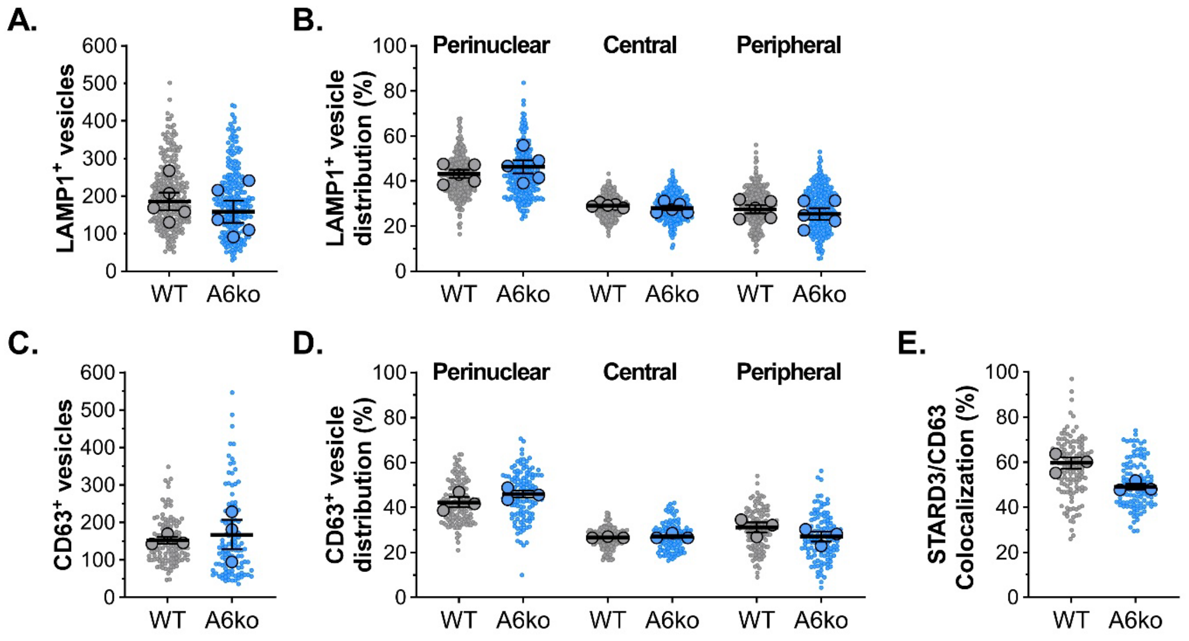

Figure S4. Quantification of LAMP1- and CD63-positive vesicle number and distribution in WT and ANXA6ko HeLa cells. Quantification of LAMP1- and CD63-positive vesicle number (A, C), distribution (B, D) and CD63/STARD3 colocalization (E) in WT and ANXA6ko HeLa cells (n > 30 cells of 5 independent measurements). Data is represented as mean ± SEM. Statistical significance was calculated using a paired Student t-test.

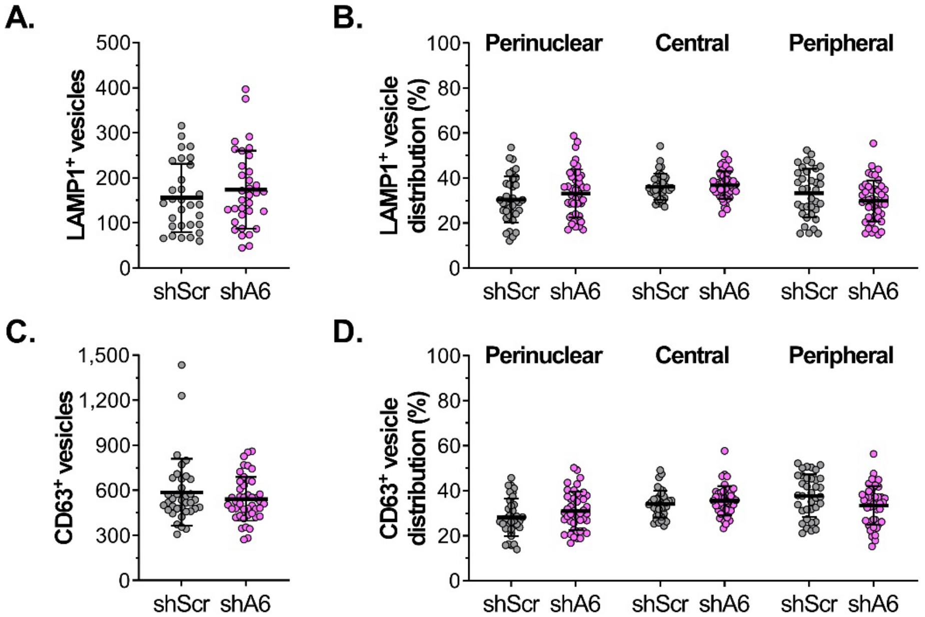

Figure S5. Quantification of LAMP1- and CD63-positive vesicle number and distribution in shScramble and shANXA6 RPE cells. Quantification of LAMP1- and CD63-positive vesicle number (A, C) and distribution (B, D) in shRNA Scramble and ANXA6 infected RPE cells (n > 30 cells, 2 independent measurements). Data is represented as mean ± SD. Statistical significance was calculated using Student t-test.

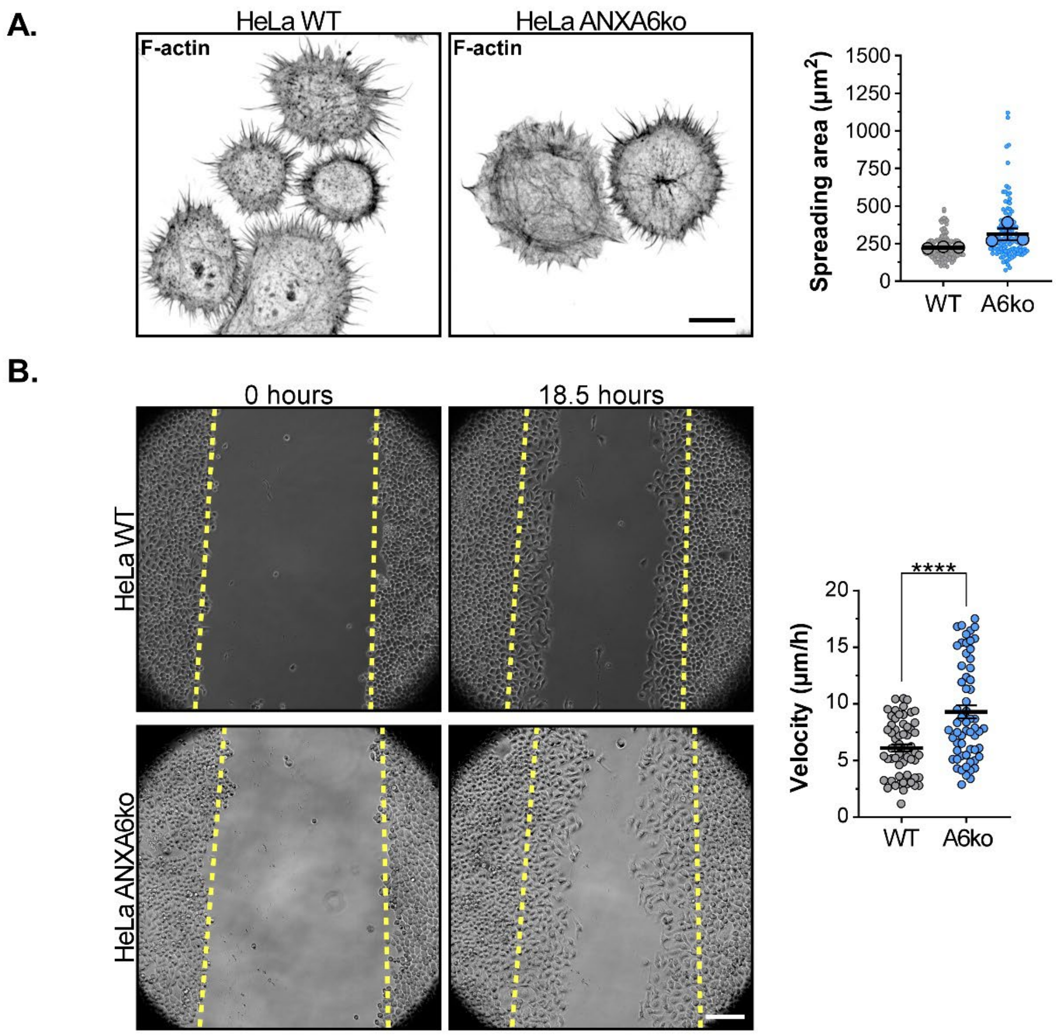

Figure S6. ANXA6 deletion increases cell motility without affecting spreading. (A) F-Actin staining and quantification of cell spreading area (n > 30 cells from 3 independent measurements). Scale bar = 10 mm. (B) Wound healing assay and quantification of migration velocities in WT and ANXA6ko HeLa cells (n > 20 images from 3 independent measurements). Data is represented as mean ± SEM. Scale bar = 100 μm. Statistical significance was calculated using a two-tailed Mann Whitney test; **** $p < 0.0001$.

SUPPLEMENTAL TABLES

Supplementary Table S1: The 18 common proteins of the three interactomes studied: ANXA6, STARD3 WT and STARD3 A6ko.

Gene	Description
TUBB4A	beta-tubulin (MT)
PHGDH	Phosphoglycerate dehydrogenase (cytosol)
HSP90AA1	Heat shock protein HSP 90 alpha (various locations)
CDK1	Cyclin-dependent kinase 1 (various locations)
PSMD11	Proteasome
SUCLA2	Succinate-CoA ligase subunit beta (mitochondria)
HSP90B1	Endoplasmin (ER)
YWHAQ	14-3-3 theta (cytosol)
TIMM44	Mitochondrial translocase TIM44
DYNC1LI2	Cytoplasmic dynein (MT)
NOTCH2	Neurogeniclocus notch homolog protein 2
STK26	Serine/threonine-protein kinase 26 (cytosol, Golgi, cytoskeleton)
SPTBN1	Spectrin-beta-chain (ABP)
ABRAXAS2	BRISC complex subunit Abraxas 2 (cytosol, cytoskeleton)
RAB27B	Ras-related protein RAB27B (lysosomal exocytosis; exosome release)
SCYL1	N-terminal kinase-like protein (Golgi architecture and secretion; mTORC1 substrate)
GSK3A	Glycogen synthase kinase-3 alpha (cytosol; various locations)
TDRD3	Tudor domain-containing protein 3 (cytosol)

Supplementary Table S2: Membrane contacts site associated proteins in the three studied interactomes: ANXA6, STARD3 WT and STARD3 A6ko.

MCS-associated (bona fide) proteins<br>Proximity-dependent biotinylation assay			
**Cell compartment**	**AnxA6**	**STARD3**	**STARD3-A6ko**
**Mitochondria**	VDAC1	FKBP8	FKBP8
	VDAC3	IMMT (MIC60)	IMMT (MIC60)
	ATAD3A	SAMM50	SAMM50
	APOOL (MIC27)	SLC25A6 (ANT)	SLC25A6 (ANT)
	CHCHD3 (MIC19)	TIMM44	TIMM44
	SNCA	OPA1	OPA1
	TOMM70	AKAP8	AKAP8
	TIMM44	CHCHD3 (MIC19)	VDAC3
	SLC25A24	TSPO	TOMM22
	MICU1	LETM1	TIMM23
		SLC25A24	ATAD3A
		MFN1	SLC25A4
		CPT1A	IDH2
		ACSL1	TSPO (low pvalue)
**ER**	VAPB	ESYT1	ESYT1
	CANX	BCAP31	PDK4
	INF2	VAPB	SOAT/ACAT1
	ITPR1	MOSPD2	
	ITPR3 (IP3R)	TMED9	
	SPAST	ACAT1/SOAT	
	TEX2	CANX	
	RAB3GAP2	PDIA6	
	CDK5		
	GRAMD2B		
	ELOVL1		
	ATP2A2		
	PI4KA		
	PI4KB		
**Endosomes**	VPS13C	STARD3 (bait)	STARD3 (bait)
	SNX2 (FFAT)	PDCD6 (ALG-2)	PDCD6 (ALG-2)
	PIKFYVE	VPS35	VPS35
	RAB14	SNX5	PDCD6IP (ALIX)
	TSG101	TSG101	LAMTOR3
	CHMP2B	LAMTOR4	LAMTOR4
	CHMP1B	RELCH	CORO1C
	OSBP (FFAT)		
	ORP1L (FFAT)		
	ORP9 (FFAT)		
	ORP10		
	RPTOR		
	VARP		
	CORO1C		
	ARL8B		
**PM**	ITGB1	ESYT1	ESYT1
	SYTL4	SYTL4	ESYT2
**Other (cytosol)**	TBC1D5	GSK3A	TBC1D5
	ANXA4		S100A8
	ANXA11		GSK3A
	ANXA6 (bait)		
	TTC1 (FFAT)		
	STK3		

	ORP2
	ORP6
	HSPA9 (GRP75)
	GSK3A

Supplementary Table S3: Cytoskeletal associated proteins in Annexin A6 interactome

**Actin binding proteins (ABPs)**	
**Gene**	**Description**
PFN2	Profilin-2
TMSB4X	Thymosin beta-4
SPTBN1	Spectrin beta chain, non erythrocytic 1
FLNA	Filamin A
EHD2	EH domain-containing protein 2
INF2	Inverted formin-2
MSN	Moesin (ERM)
RDX	Radixin (ERM)
EZR	Ezrin (ERM; regulator of lysosome function; interact with Vps11, HOPS complex)
NF2	Merlin
DBNL	Drebrin-like protein
CARMIL1	F-actin-uncapping protein
CARMIL2	Capping protein, Arp2/3 and myosin-I linker protein 2
CORO1C	Coronin-1C
DMD	Dystrophin
TRIOBP	TRIO and F-actin binding protein
ADD1	Alpha-adducin
ADD2	Gamma-adducin
PDLIM7	PDZ and LIM domain protein 7
PDLIM1	PDZ and LIM domain protein 7 (aka, CLP-36; membrane trafficking)
ARPC5	Actin-related protein 2/3 complex subunit 5
DIAPH1	Protein diaphanous homolog 1
BAIAP2L1	Brain-specific angiogenesis inhibitor 1-associated protein 2-like protein 1
BAIAP2	Brain-specific angiogenesis inhibitor 1-associated protein 2
ENAH	Protein enabled homolog
ANXA6	Annexin A6 (bait)
ACTB	Actin, cytoplasmic 1 (low pvalue)

**Myosins + Unconventional Myosins + Tropomyosins**	
**Gene**	**Description**
MYO9B	Unconventional myosin-IXb
MYO5A	Unconventional myosin-Va
MYO1B	Unconventional myosin-Ib
MYO18A	Unconventional myosin-XVIIIa (microvilli)
MYH10	Myosin-10 (filopodia)
TPM2	Tropomyosin beta chain

**Microtubule and Microtubule associate proteins (MAPs)**	
**Gene**	**Description**
MACF1	Microtubule-actin cross-linking factor 1, isoforms 1/2/3/5
MTCL1	Microtubule cross-linking factor 1
TUBB4A	Tubulin beta-4A chain
KIF5B	Kinesin-1 heavy chain
KLC2	Kinesin light chain 2
KIF13A	Kinesin-like protein KIF13A
KIF1B	Kinesin-like protein KIF1B
KLC1	Kinesin light chain 1

KLC4	Kinesin light chain 4
KIF11	Kinesin-like protein KIF11
KIF16B	Kinesin-like protein KIF16B
DYNC1H1	Cytoplasmic dynein 1 heavy chain 1
DYNC2I1	Cytoplasmic dynein 2 intermediate chain 1
DYNC1LI2	Cytoplasmic dynein 1 light intermediate chain 2
DCTN4	Dynactin subunit 4
AURKA	Aurora kinase A

Other cytoskeletal-related proteins

**Gene**	**Description**
PLEKHG3	Pleckstrin homology domain-containing family G member 3
PHLDB2	Pleckstrin homology-like domain family B member 2
FARP1	FERM, ARHGEF and pleckstrin domain-containing protein 1
PLEK2	Pleckstrin-2
FARP2	FERM, ARHGEF and pleckstrin domain-containing protein 2
PLEKHA6	Pleckstrin homology domain-containing family A member 6
PHLDA1	Pleckstrin homology-like domain family A member 1
PLEKHA5	Pleckstrin homology domain-containing family A member 5
ITSN2	Intersectin-2
ITSN1	Intersectin-1
CKAP5	Cytoskeleton-associated protein 5
CKAP2	Cytoskeleton-associated protein 2

Supplementary Table S4: Cytoskeletal associated proteins in the STARD3 WT interactome

**Actin binding proteins (ABPs)**	
**Gene**	**Description**
ACTB	Actin. cytoplasmic 1 (pvalue=1.113)
VASP	Vasodilator-stimulated phosphoprotein
FLNA	Filamin-A
EZR	Ezrin (binds to EGFR and is a regulator of lysosomal function)
MSN	Moesin
CTTN	Src substrate cortactin
SPTBN1	Spectrin beta chain. non-erythrocytic 1
DMD	Dystrophin
WIPF2	(WIRE)
STK26	Serine/threonine-protein kinase 26 (aka MST4)
ESYT1	Extended synaptotagmin-1
CDK1	Cyclin-dependent kinase 1 (phosphorylates SEPTIN9)
SYAP1	Synapse-associated protein 1
CFL1	Cofilin-1

**Tubulin and Microtubules associated proteins (MAPs)**	
**Gene**	**Description**
TUBB4B	Tubulin beta-4B chain
TUBB4A	Tubulin beta-4A chain
TUBA1C	Tubulin alpha-1C chain
TUBG1	Tubulin gamma-1 chain
TBCD	Tubulin-specific chaperone D
DYNC1H1	Cytoplasmic dynein 1 heavy chain 1
DYNC1LI2	Cytoplasmic dynein 1 light intermediate chain 2
DNAAF5	Dynein axonemal assembly factor 5

**Other proteins**	
**Gene**	**Description**
VIM	Vimentin
SEPTIN7	Septin-7
SEPTIN11	Septin-11
SEPTIN10	Septin-10

Supplementary Table S5: Cytoskeletal associated proteins in the STARD3-ANXA6ko interactome

**Actin binding proteins (ABPs)**	
**Gene**	**Description**
SPTBN1	Spectrin beta chain, non-erythrocytic 1
GSN	Gelsolin
ACTG1	Actin, cytoplasmic 2
ACTB	Actin, cytoplasmic 1
CAPN1	Calpain-1 catalytic subunit
TTN	Titin
CAPZB	F-actin-capping protein subunit beta
LIMA1	LIM domain and actin-binding protein 1
LIMCH1	LIM and calponin homology domains-containing protein 1
ACTA1	Actin, alpha skeletal muscle
ACTR3	Actin-related protein 3
ACTBL2	Beta-actin-like protein 2
AFAP1	Actin filament-associated protein 1
ACTN4	Alpha-actinin-4
ACTL6A	Actin-like protein 6A
TAGLN2	Transgelin-2
RAI14	Ankycorbin
SVIL	Supervillin
SPECC1L	Cytospin-A
DSP	Desmoplakin
CORO1C	Coronin-1C
CORO1B	Coronin-1B
TMOD3	Tropomodulin-3
SCIN	Adseverin
FSCN1	Fascin
CKAP4	Cytoskeleton-associated protein 4

**Myosins and Tropomyosins**	
**Gene**	**Description**
MYH9	Myosin-9 (myosin IIa) (validated: co-IP with STARD3)
MYH10	Myosin-10 (myosin IIb) (stress fibers)
MYO1C	Unconventional myosin-Ic
MYO1E	Unconventional myosin-Ie
MYO1B	Unconventional myosin-Ib
MYO18A	Unconventional myosin-XVIIIa
MYO6	Unconventional myosin-VI (microvilli)
MYO5A	Unconventional myosin-Va (STARD3 in peripheral LE/Lys)
MYO1D	Unconventional myosin-Id
MYH14	Myosin-14 (microvilli)
MYO5C	Unconventional myosin-Vc
MYL6	Myosin light polypeptide 6
MYL6B	Myosin light chain 6B
MYH7	Myosin-7
TPM4	Tropomyosin alpha-4 chain
TPM3	Tropomyosin alpha-3 chain
TPM1	Tropomyosin alpha-1 chain

Tubulin and Microtubule-associated proteins (MAPs)	
**Gene**	**Description**
TUBB4A	Tubulin beta-4A chain
TUBA1A	Tubulin alpha-1A chain
TUBB6	Tubulin beta-6 chain
TUBB8B	Tubulin beta 8B
TUBAL3	Tubulin alpha chain-like 3
MARK3	MAP/microtubule affinity-regulating kinase 3
DYNC1LI2	Cytoplasmic dynein 1 light intermediate chain 2
DCTN1	Dynactin subunit 1
DYNC1I2	Cytoplasmic dynein 1 intermediate chain 2
KIF5B	Kinesin-1 heavy chain
MAP7	Ensconsin (interacts with KIF5B)

Other proteins	
**Gene**	**Description**
SEPTIN2	Septin-2
SEPTIN9	Septin-9 (Lys positioning; association with LD)
SEPTIN6	Septin-6
SEPTIN8	Septin-8
